# Functional areas shape indoor microbial structure and potential risks in university dormitories

**DOI:** 10.3389/fmicb.2025.1604064

**Published:** 2025-07-15

**Authors:** Huaiyu Cao, Hanbing Ye, Yucheng Tian, Jinhong Zhang, Yongchao Xie, Yuan Chen, Qiuyue Mo, Song Huang, Yiqi Tao, Tang Liu

**Affiliations:** ^1^School of Architecture and Urban Planning, Shenzhen University, Shenzhen, China; ^2^School of Environment and Energy, Peking University Shenzhen Graduate School, Shenzhen, China; ^3^Environmental Microbiome Engineering and Innovative Genomics Laboratory, College of Chemistry and Environmental Engineering, Shenzhen University, Shenzhen, China; ^4^Department of Life Sciences and Medicine, University of Science and Technology of China, Hefei, China; ^5^State Key Laboratory of Subtropical Building and Urban Science, Shenzhen University, Shenzhen, China; ^6^Center for Human-oriented Environment and Sustainable Design, Shenzhen University, Shenzhen, China

**Keywords:** college dormitory, microbial community, spatial variations, pathogenicity, health risk

## Abstract

Exposure to indoor microbes, particularly potential pathogens, poses significant health risks to occupants. While the indoor microbiome has been extensively studied in various settings, its spatial distribution in university dormitories within hot and humid climates remains poorly understood. In this study, 56 samples were collected from four functional areas (air conditioning, sink, toilet, and floor) in student dormitories in Shenzhen, China. 16S rRNA gene sequencing revealed that the indoor microbial communities were predominantly composed of human-associated genera such as *Kocuria, Corynebacterium*, and *Staphylococcus,* with marked compositional differences among functional zones. FAPROTAX predictions further identified 74 potential human pathogens, mainly linked in literature to the risks of nosocomial infections and pneumonia. Notably, a significant portion of these pathogens belongs to the genus *Acinetobacter*, with elevated concentrations found in air conditioning systems, suggesting their potential as reservoirs of clinically relevant microbes. Environmental variations across room functional areas significantly influenced the composition profile of the microbiome, while the impact of occupant characteristics appeared negligible. Key environmental factors, particularly temperature, played a major role in shaping both microbial and pathogen dynamics. Floor surfaces were identified as key microbial hotspots, exhibiting complex microbial networks that interacted strongly with communities from other functional areas. This underscores the floor’s vital role in maintaining connectivity within the indoor environment. The assembly processes of indoor microbial and predicted pathogen communities were both dominated by stochastic processes, with the former primarily governed by dispersal limitations and the latter by ecological drift. Overall, this study provides critical insights into the spatial distribution, environmental drivers, and assembly mechanisms of microbial and pathogen communities in university dormitories, contributing to future assessments of indoor microbial exposure and hygiene management.

## 1 Introduction

Modern human lifestyles tether us to indoor environments for more than 90% of our daily routines (Klepeis et al., 2001), significantly magnifying the profound impact of indoor microbial exposure on individual health and well-being (Sun et al., 2022a). This trend has spurred extensive research into the characterization of indoor microbial compositions, succession and ecology to better understand the microbial interactions between humans and their built environment (Adams et al., 2015; Klassert et al., 2021). In the last decades, while significant attention has been devoted to indoor microbiota in diverse settings including public transportation (Leung et al., 2014; Grydaki et al., 2021), hospitals (Oberauner et al., 2013), and classrooms (Sun et al., 2022b), a notable gap remains in our perception of on-campus dormitories. Unlike conventional living spaces, most dormitories in China are compact apartments characterized by high occupancy and limited space. These environments, which bring together young individuals for extended periods, are likely to significantly influence students’ health (Fu et al., 2021).

Microbial genome sequencing has revealed that indoor environments harbor a myriad of microorganisms, including diverse human-associated taxa such as *Propionibacterium*, *Corynebacterium*, and *Staphylococcus*, primarily originating from outdoor air and household occupants (Leung et al., 2014; Wilkins et al., 2016; Richardson Richardson Miles et al., 2019; Young et al., 2023). Building factors—such as design, ventilation, and indoor decoration—are crucial determinants that contribute to shaping indoor microbial communities (Sharpe et al., 2020; Amin et al., 2023; Zhang et al., 2023; Hoisington et al., 2023), whereas environmental parameters, including temperature, humidity and PM2.5 further influence microbial concentrations (Ye et al., 2021a, 2021b). Of particular interest, factors including building age and vacuum dust weight have been shown to impact microbial composition in dormitories (Fu et al., 2020). Given this, functional areas within the same room were postulated to be exposed to drastically different microbial communities due to variations in functional areas and building elements. However, this hypothesis was supported by comparatively few studies (Dunn et al., 2013; Zhou et al., 2023), leaving gaps in our understanding of the comprehensive distribution of indoor microorganisms across different locations.

Although predominantly present indoor microorganisms are not associated with immediate health concerns, certain groups may be linked to infection risks (Leung et al., 2019). Early microbiome-health studies have widely reported the association between indoor microbiome exposure and the prevalence of immune diseases such as asthma, rhinitis and eczema (Kim et al., 2018; Fu et al., 2023). For instance, taxa from distinct phylogenetic classes and derived habitats, identified through 16S rRNA amplicon sequencing, exhibit varying impacts on asthma symptoms among residents in on-campus dormitories (Fu et al., 2021). The indoor environment may further amplify the disease risks posed by pathogens (Li et al., 2021). Millions of microorganisms, fragments and microbial metabolites present in indoor air can facilitate disease transmission when inhaled into the human respiratory tract, potentially inducing allergic and inflammatory reactions (Qian et al., 2012; Chen Y. et al., 2024). More alarmingly, all reported outbreaks of SARS-CoV-2 involving three or more individuals occurred indoors, further underscoring the critical link between indoor environments and disease transmission (Allen and Marr, 2020; Mutsch et al., 2022). Therefore, in-depth research into potentially pathogenic microorganisms with unexplored functions and impacts in enclosed environments is imperative for enhancing indoor environmental quality and public health.

Despite the growing interest in dormitory microbiomes, the impact of microenvironmental variations across different functional areas within the room on microbial community structures and the prevalence of potentially pathogenic microorganisms remains inadequately characterized. In this study, we explored the microbial diversity and potential pathogens within 18 university dormitories in Shenzhen, Guangdong Province, China. Microbial samples were collected from four distinct functional areas, including the air conditioning (AC), the sink (SN), the toilet (WC), and the floor surface (WL). Utilizing 16S rRNA high-throughput sequencing, we delved into the intricate community patterns, assemblages, and networks of the indoor bacterial communities at each site. Specifically, we aimed to: (1) elucidate the taxonomic diversity of indoor microbiota across various dormitory functional areas by revealing the roles of environmental factors and assembly processes in shaping these communities, including potential pathogens; (2) identify general and location-specific relationships between dormitory functional areas and their associated microbiota to explain the connectivity among them; (3) evaluate the presence, biogeography, and transmission processes of potential pathogen communities within dormitories, while cautiously considering their health relevance in light of the limitations of 16S rRNA sequencing. Our findings will provide a theoretical foundation for enhancing the comfort of on-campus dormitories and promoting occupant safety and health.

## 2 Materials and methods

### 2.1 Study design and sample collection

**Figure 1:**
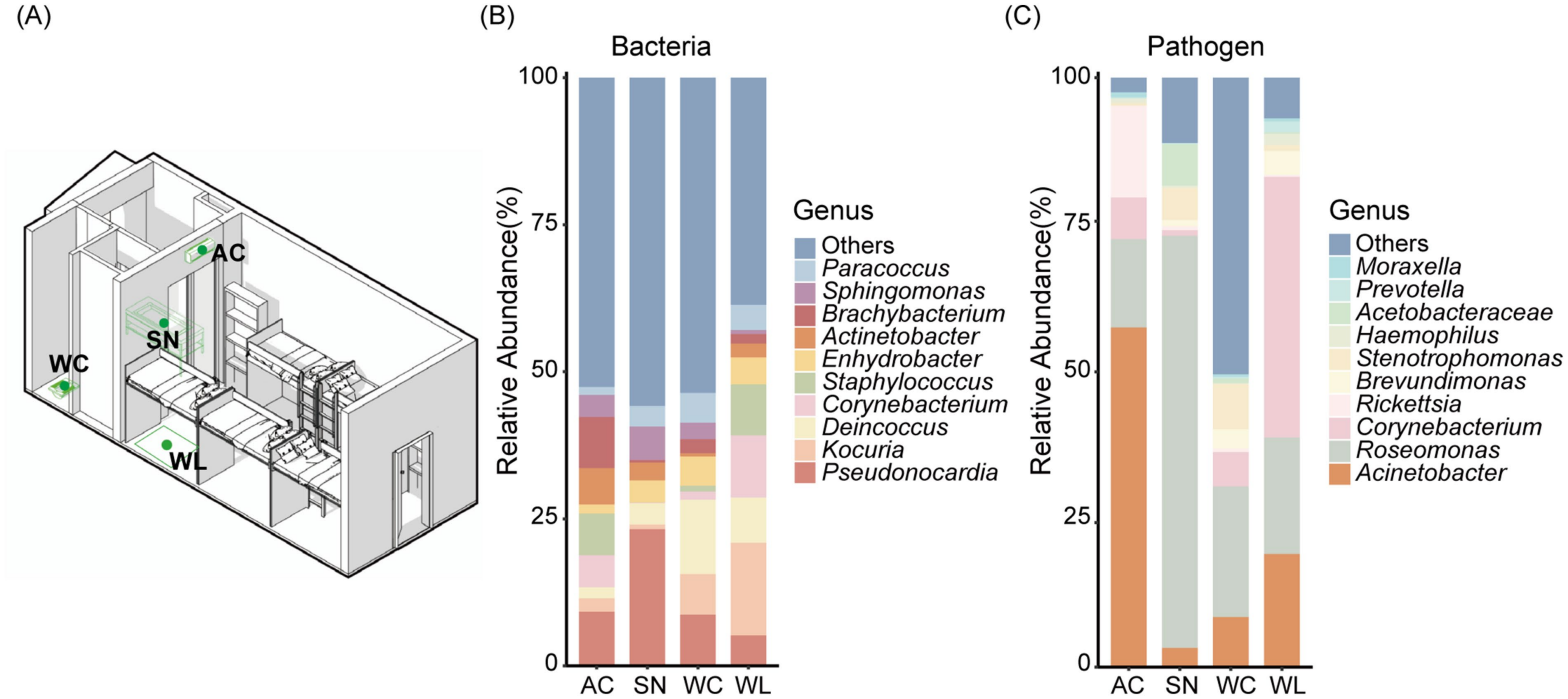
Dormitory sampling points and microbial composition. **(A)** Four functional areas within the dormitory: air conditioning (AC), sink (SN), toilet (WC), and floor surface (WL). **(B)** Genus-level bacterial composition across the different functional areas within the dormitory. **(C)** Genus-level composition of potential pathogens in various dormitory locations.

A total of 18 student dormitories in Guangdong, China, were chosen as sampling locations. Sampling was conducted at four distinct functional areas within each room: the air conditioning (AC), the sink (SN), the toilet (WC), and the floor (WL) (Figure 1A). These site types were selected based on their representation of typical functional areas within dormitories and their distinct environmental characteristics (e.g., humidity, temperature, airflow, and frequency of human contact), which are expected to influence microbial biomass and community structure (Supplementary Tables 1, 2). Sterile sampling swabs and Beckman Coulter sampling tubes were used to collect dust microbial samples. All samples were immediately placed in sterile bags and stored at −20°C in a freezer until further analysis, ensuring the preservation of sample integrity.

In addition to microbial sampling, environmental parameters including temperature (Temp), relative humidity (RH), air velocity (Va), floor level (Height), and balcony orientation (Orientation) were measured *in situ* for each room using a TESTO 480 Indoor Comfort Meter and a JT-IAQ Indoor Thermal Environment and Air Quality Tester.

### 2.2 DNA extraction, sequencing and bioinformatic analysis

DNA samples were extracted using the Fast DNA Spin Kit for Soil (MP Biomedicals, United States) following the authoritative instructions. The purity and concentration of the DNA were measured using a NanoDrop One spectrophotometer (Thermo Fisher Scientific, MA, United States) and DNA quality was assessed through 1% agarose gel electrophoresis. The V3-V4 hypervariable region of the 16S rRNA gene was amplified with the primer set 338F/806R (338F: 5′-ACTCCTACGGGAGGCAGCA-3′; 806R: 5′-GGACTACHVGGG TATCTAAT-3′) (Caporaso et al., 2011; Dai et al., 2022). Libraries were prepared using the NEBNext® Ultra™ II DNA Library Prep Kit for Illumina® (New England Biolabs, MA, United States) according to the manufacturer’s instructions. Due to failures in meeting the standards for library construction and the absence of toilet facilities in some dormitories, a total of 56 samples (16 AC samples, 17 SN samples, 5 WC samples, and 18 WL samples) were ultimately sequenced on the Illumina Nova6000 platform, yielding 250 bp paired-end reads at Guangdong Magigene Biotechnology Co., Ltd. (Guangzhou, China).

Raw 16S rRNA reads of each sample were quality-filtered, trimmed, and screened using fastp (version 0.14.1)^1^ and cutadapt.^2^ After quality control, high-quality sequences were processed into amplicon sequence variants (ASVs) using the Deblur plugin in the QIIME2 platform, with taxonomic classification performed with the Silva database (v. 138) (Quast et al., 2012). To ensure comparability among samples and mitigate biases arising from discrepancies in sequencing depth, sequences were standardized to an even depth based on the sample with the lowest number of reads (21,710 sequences per sample). Rarefaction curves confirmed that this threshold retained sufficient diversity for reliable downstream analysis (Supplementary Figure S1). Functional Annotation of Prokaryotic Taxa (FAPROTAX), based on taxonomic affiliation and curated literature, was utilized to annotate the potential pathogenicity of microbial taxa (Louca et al., 2016; Labouyrie et al., 2023). ASVs assigned to at least one potentially pathogenic group were extracted to construct a community comprising potential pathogenic bacteria.

### 2.3 Statistical analysis

The *vegan* package in R (version 4.4.0) was utilized to calculate ASV richness and the Shannon index for the measured microbial communities (Oksanen et al., 2022). Statistical differences in ASV richness across various indoor functional areas were analyzed using the non-parametric Kruskal–Wallis test and Dunn’s *post hoc* test using the R package *dunn.test*. Principal Coordinate Analysis (PCoA) based on Bray-Curtis distance was conducted to assess the *β*-diversity of bacterial communities among samples. Analysis of similarity (ANOSIM) was applied to test the significance of the community differences across various functional areas at a significance level of *p* < 0.05. Furthermore, β-diversity was partitioned into turnover and nestedness components using the R package *adespatial* according to the method proposed by Baselga (Baselga, 2010). Linear Discriminant Analysis (LDA) coupled with effect size analysis (LEfSe) was performed to identify statistically different biomarkers across dormitory functional areas using the R package *microeco*. To address group size imbalance, a subsampling procedure was conducted by randomly downsampling the larger SN group to match the sample size of the WC group. Taxa with LDA scores greater than 4 were visualized using bar plots.

Canonical Correspondence Analysis (CCA) was carried out using the *vegan* package to reveal the effects of environmental parameters on microbial and potentially pathogenic communities. Mantel test was conducted to detect the correlation between the environmental variables and microbial communities using the *linkET* R package. The contributions of five ecological processes (homogeneous selection, heterogeneous selection, homogeneous diffusion, diffusion limitation, non-dominated process) to the community structure was explored using the iCAMP package (Stegen et al., 2013; Zhou and Ning, 2017; Wang et al., 2020).

Co-occurrence networks of microbial and potentially pathogenic bacteria were constructed from ASVs present in at least 20% of samples based on robust correlations with Spearman’s correlation coefficients > 0.6 and false discovery rate-corrected *p* < 0.01(Guo et al., 2022; Yang et al., 2022), using the *WGCNA* package. The *Hmisc* and *igraph* packages in R were then utilized to compute the node and edge files of the network graph, which were subsequently visualized using the interactive platform Gephi (v. 0.10.1).^3^ The contributions of different sampling functional areas to the community composition of the floor were predicted with fast expectation–maximization for microbial source tracking (FEAST) (Shenhav et al., 2019).

## 3 Results

### 3.1 Taxonomic diversity of the dormitory bacterial and pathogenic communities

A total of 8,243 Amplicon Sequence Variants (ASVs) were recovered across 56 samples. The dataset encompassed bacteria from 28 phyla, 76 classes, 202 orders, 380 families and 1,052 genera, covering a broad taxonomic range and a substantial portion of uncultivated microorganisms. At the phylum level, the dormitory dataset was dominated by Actinobacteriota (38.79%), followed by Proteobacteria (32.41%) and Firmicutes (7.39%) (Supplementary Figure S2A). Actinobacteriota was also predominant in AC (58.16%) and WL (43.94%), while the abundant phyla at SN differed dramatically, with the top three phyla being Proteobacteria (50.42%), Actinobacteriota (22.35%), and Patescibacteria (9.20%) (Supplementary Figures S2C, S3). At the genus level, *Pseudonocardia* was the common dominant genera widely distributed across all four sampling functional areas. *Kocuria* (15.76%) and *Corynebacterium* (10.56%) featured prominently in WL, while *Deinococcus* (12.61%) dominated in WC and *Brachybacterium* (8.69%) dominated in AC (Figure 1B). These results underscored the substantial differences of community composition across functional areas within the dormitory.

To further assess the health risks within the living spaces, the presence of potential human pathogenic bacteria was also investigated using FAPROTAX. A total of 372 ASVs were predicted as potential bacterial pathogens, accounting for 4.5% of all ASV numbers and 5.24% of total abundance. Among these, 74 ASVs were identified to be associated with humans, spanning 6 potential disease groups. The most prevalent group was human nosocomial pathogens (HumPN), with a relative abundance (RA) of 6.39%, followed by human pneumonia pathogens (HumPP) at 5.12%. Other identified categories included human septicemia pathogens (HumPS, RA = 1.87%), human diarrhea pathogens (HumPD, RA = 0.01%), human gastroenteritis pathogens (HumPG, RA = 0.01%), and human meningitis pathogens (HumPM, RA = 0.01%) (Supplementary Table 3). HumPN and HumPP exhibited significant variations across functional areas. Specifically, HumPN was notably enriched in WL compared to WC (Emmeans test, *p* < 0.001), while HumPP showed a higher abundance in WC compared to AC and SN (*p* < 0.05; Supplementary Figure S4).

The potential human-associated pathogen community was assigned to 5 phyla, with the dominant phyla being Proteobacteria (85.25%) and Actinobacteria (14.58%) (Supplementary Figure S2B). Pathogen phyla distribution showed that Proteobacteria comprised the majority of sequences in samples from AC (95.73%), SN (88.86%), and WC (97.98%), whereas some variation was observed in WL, where Actinobacteriota demonstrated a notable presence (41.46%) (Supplementary Figures S2D, S5). Taxonomic annotation results further revealed significant changes in genus-level relative abundances across functional areas (Supplementary Table 4), with *Acinetobacter*, *Corynebacterium* and *Oligella* being the dominant genera in AC, WL, and WC, respectively (Figure 1C).

**Figure 2:**
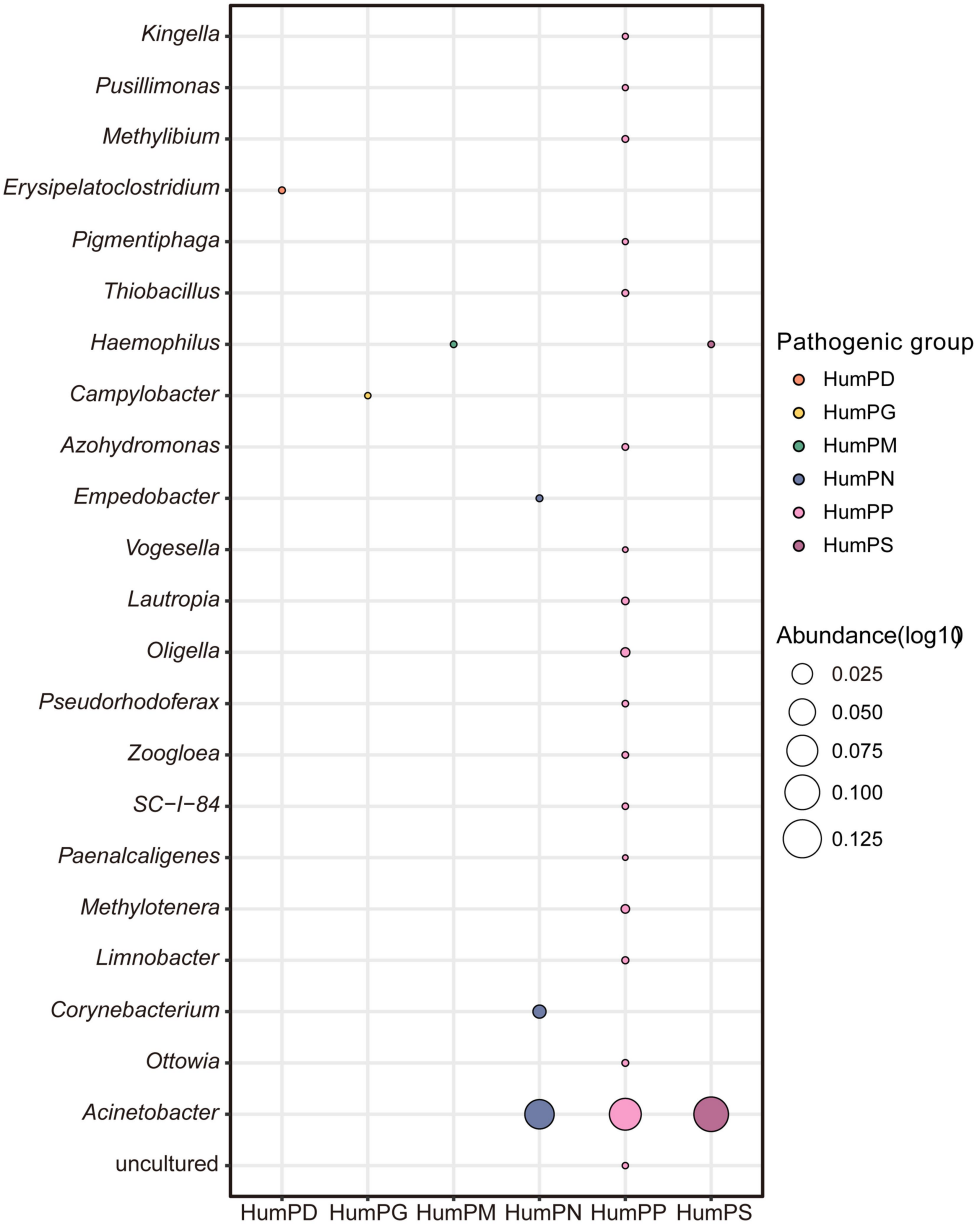
Relationships between human pathogen groups and bacterial genera. Circle size represents abundance, and different colors indicate distinct human-associated pathogenic functional groups. Abundance values were transformed using the ln(x + 1) function. (AniP, animal parafunctional areas or symbionts; HumPA, human pathogens all; HumPN, human pathogens nosocomial; IntCelP, intracellular parafunctional areas; HumPP, human pathogens pneumonia; PlaP, plant pathogens; HumPS, human pathogens septicemia; InvP, invertebrate parafunctional areas; HumPD, human pathogens diarrhea; HumPG, human pathogens gastroenteritis; HumPM, human pathogens meningitis).

Taxonomically distinct microbial communities exhibited varying contributions to different classes of potential human pathogens (Figure 2; Supplementary Figure S6). At the phylum level, Proteobacteria exhibited markedly high abundance across multiple human-associated pathogenic functions, particularly HumPN and HumPS. In addition to Proteobacteria, HumPN also received notable contributions from Actinobacteria and Bacteroidota (Supplementary Figure S6). At the genus level, HumPP was broadly distributed, with *Acinetobacter*, a member of Proteobacteria, showing the highest abundance. Notably, *Acinetobacter* was also the dominant contributor to HumPN and HumPS, highlighting its key role as a potential pathogen in indoor environments. Other genera, including *Erysipelatoclostridium*, *Haemophilus* and *Campylobacter*, were primarily associated with HumPD, HumPM, and HumPG, respectively (Figure 2). Together, these findings reveal distinct taxonomic contributions to the pathogenic potential of indoor microbiomes.

### 3.2 Microbial distribution and diversity across dormitory functional areas

**Figure 3:**
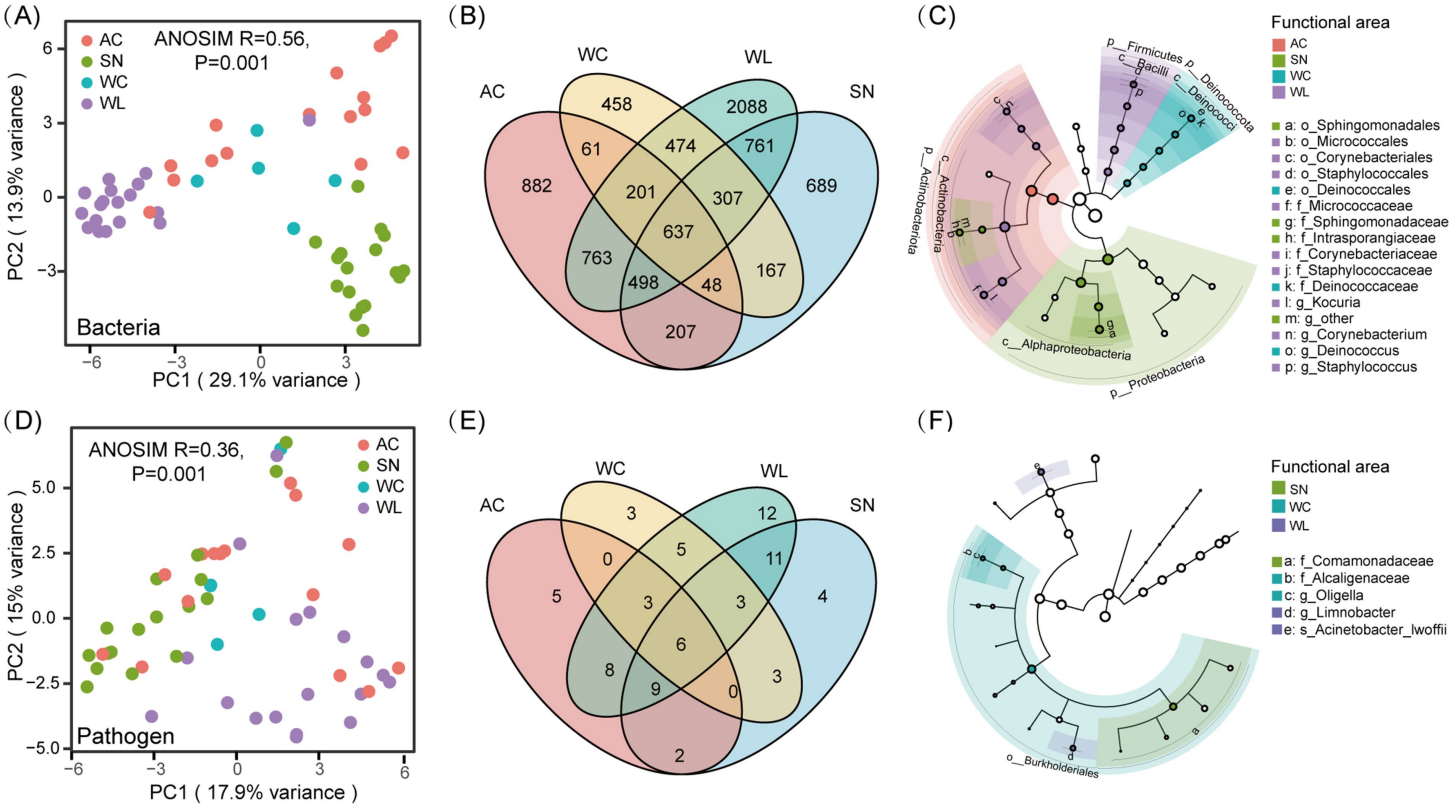
Diversity patterns and species composition of the overall bacterial community and potential pathogens in different functional areas of the dormitory. **(A)** PCoA analysis of bacterial communities across different functional areas; **(B)** Venn diagram showing shared species; **(C)** LEfSe analysis of bacterial communities; **(D)** PCoA analysis of potential pathogen communities across different functional areas; **(E)** Venn diagram showing shared species of potential pathogens; **(F)** LEfSe analysis of potential pathogen communities.

Microbial communities, together with human pathogens, exhibited distinct patterns of *α*-diversity across the four dormitory functional areas. WL showed the highest ASV richness for both microbial and potential pathogenic bacteria, significantly exceeding the other three functional areas (*p* < 0.01; Supplementary Figure S7A). The Shannon index further confirmed a more diverse and evenly distributed microbiome in WL, with significant differences observed compared to AC (*p* < 0.01; Supplementary Figure S7B). To further compare bacterial community structures across functional areas, Principal Coordinates Analysis (PCoA) was conducted using a Bray-Curtis distance matrix (Figure 3A). The first two PCoA axes explained 43% of bacterial community variation, exhibiting a moderate yet significant dissimilarity across functional areas, which was supported by ANOSIM (Supplementary Figure S8A, R = 0.56, *p* = 0.001). Minor differences in microbial community composition were observed between WC and AC, whereas samples from WL and SN were well separated. To compare, the *β*-diversity across different dormitory rooms failed to present clear clustering (Supplementary Figure S9), indicating that the different functional areas of dormitory were more appropriate for understanding microbial distribution patterns. Similarly, significant separation was observed in the pathogenic communities across functional areas (Supplementary Figure S8B, ANOSIM R = 0.36, *p* = 0.001), with samples from WL and SN exhibited greater dispersion, indicating a higher degree of community heterogeneity (Figure 3D).

Analysis of shared and unique ASVs revealed that only 7.73% were shared among all four functional areas, suggesting these ASVs exhibited stronger environmental adaptability, allowing them to survive and thrive across diverse dormitory environments (Figure 3B). The number of ASVs specific to WL (*n* = 2088) was 2 to 4 times higher than those at other functional areas, consistent with the highest microbial richness observed in WL. The Venn diagram further described the coexistence pattern of human-associated pathogens within the room, with WL containing the highest number of unique ASVs (*n* = 12; Figure 3E).

As clustering by sampling functional areas was evident in the *β*-diversity analysis, we further applied the linear discriminant analysis effect size (LEfSe) tool to identify site-specific biomarkers, with LDA scores of 4 or more presented. To mitigate the potential impact of unbalanced group sizes, we conducted a random subsampling analysis by downsampling the SN group to match the smaller WC group (Supplementary Figure S10). The specialized bacterial community at the four sampling functional areas diverged markedly, with greater numbers of species enriched at significant level in SN and WL. In SN, Proteobacteria was significantly enriched across multiple taxonomic levels, while WL showcased a prevalence of Firmicutes and WC was mainly characterized by Deinococcota from phylum to species levels (Figure 3C; Supplementary Figure S10A). In contrast to these cases where a single phylum dominated one site throughout the taxonomic hierarchy, Actinobacteriota at different taxonomic levels exhibited notable biomarker significance in functional areas. For instance, families Corynebacteriaceae and Micrococcaceae were dramatically enriched in WL group, while Intrasporangiaceae showed significant enrichment in SN group. For potential pathogenic taxa, no biomarkers in the AC group had LDA scores above 4, thus no significant pathogens were plotted for this group (Figure 3F). The significant presence of multiple Proteobacteria families, such as Comamonadaceae in SN and Alcaligenaceae in WC, indicated a specific environmental adaptation of different potentially pathogenic Proteobacteria within the dormitory (Supplementary Figure S10B).

### 3.3 Environmental drivers of bacterial community in the dormitory

**Figure 4:**
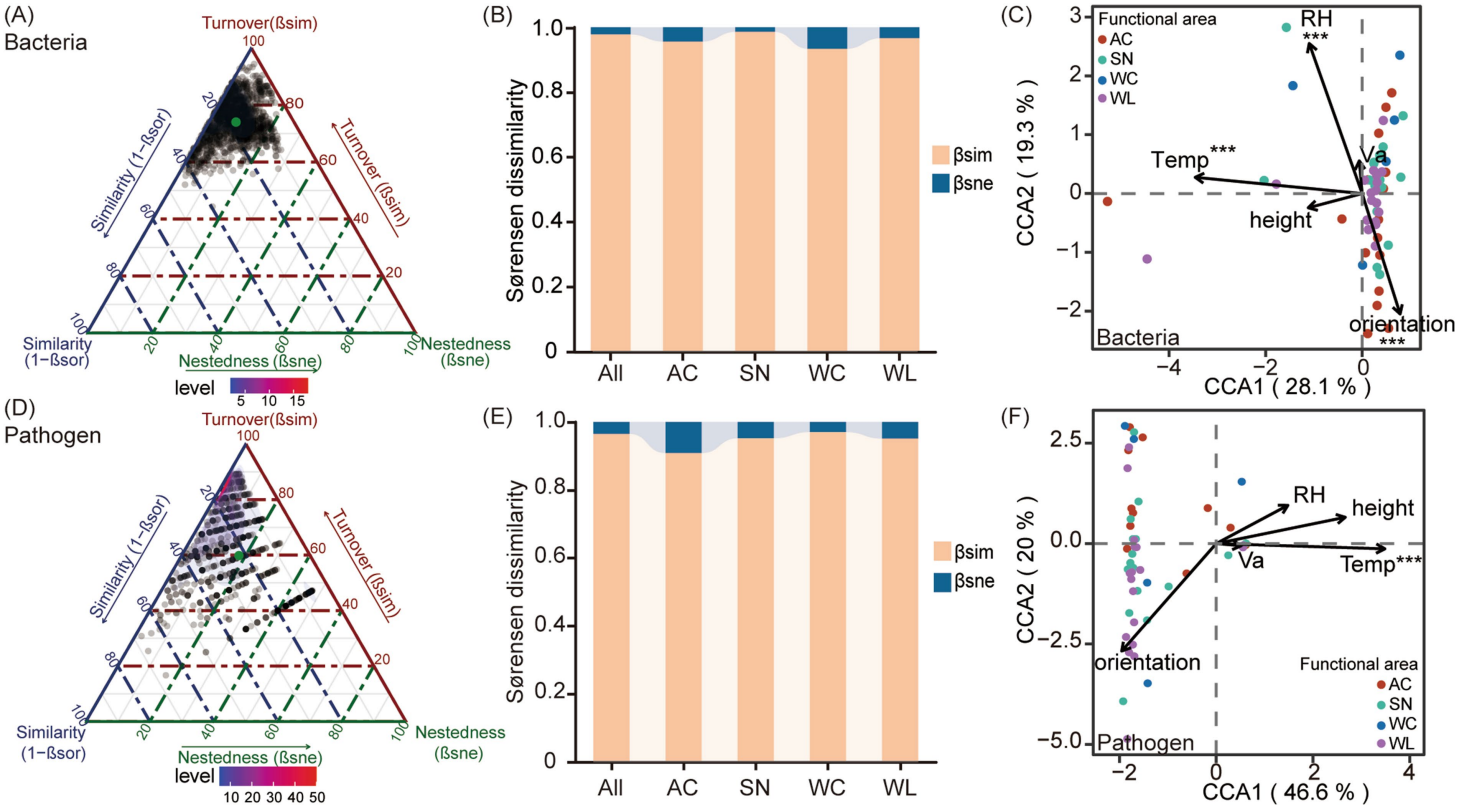
Beta diversity partitioning and correlation analysis of dormitory microorganisms and potential pathogens with environmental factors. **(A)** Ternary plot showing beta diversity partitioning for the overall bacterial community; **(B)** Proportions of different dissimilarity components in the beta diversity of the overall bacterial community; **(C)** CCA analysis of environmental factors affecting bacterial communities; **(D)** Ternary plot showing beta diversity partitioning for potential pathogen communities; **(E)** Proportions of different dissimilarity components in the beta diversity of potential pathogen communities; **(F)** CCA analysis of environmental factors affecting potential pathogen communities.

We next conducted *β*-partitioning analysis to decompose the overall β-diversity into turnover and nestedness components. According to the partitioning results, the variation in β-diversity among assemblages of the indoor microbial and potentially pathogenic community was mainly explained by the species turnover process (microorganisms: β_sim_ = 90.6%; pathogenic bacteria: β_sim_ = 85.6%) (Figures 4A,D). The high proportions of species turnover component across all dormitory functional areas further indicated that such processes dominantly structured both the microbial and the pathogenic taxa (Supplementary Figures S11, S12). Notably, the relative contribution of the nestedness component was slightly higher in AC and SN, but neither exceeded 20% (Figures 4B,E), suggesting that nestedness process remains a minor contributor to β-diversity overall.

The influences of environmental factors on the β-diversity of microbial and pathogenic communities were explored by performing CCA ordination (Figures 4C,F). The first two ordination axes explained a substantial portion of the taxonomic variation within the microbial and pathogenic communities across the 56 samples, accounting for 47.4% of the variation in microbial communities and 66.6% in pathogenic communities. Temperature (Temp, *R*^2^ = 0.82, *p* < 0.01), relative humidity (RH, *R*^2^ = 0.58, *p* < 0.01) and orientation (*R*^2^ = 0.36, *p* < 0.01) were significantly related to shifts in the overall bacterial community. In contrast, the potential pathogen community only exhibited a significant correlation with temperature (*R*^2^ = 0.94, *p* < 0.01) but showed no correlation with height, RH, air velocity (Va), or orientation (*p* > 0.05).

The CCA analysis of bacterial taxa at different sampling functional areas also revealed site-specific environmental responses. Temperature was found to be correlated with microbial community composition at most functional areas (AC, SN, and WL). Humidity was another significant factor for microbial communities in SN and WL. Additionally, the microbial community in AC responded to Va; in SN, to orientation; and in WL, to height (Supplementary Figure S13). No significant correlations were found for the microbial community in WC with any environmental factor. Similarly, for pathogen communities at functional areas, the CCA results also indicated a significant association between temperature and the pathogenic communities in AC, SN, and WL. SN samples were additionally related to levels of sunlight exposure (orientation) and height (Supplementary Figure S14). Among the correlated environmental variables, room temperature exhibited the strongest correlation with the potential pathogen community in dormitory rooms, which was further confirmed by correlation analysis using Mantel tests (Supplementary Figures S15, S16).

### 3.4 Co-occurrence patterns and their topological features in the dormitory

**Figure 5:**
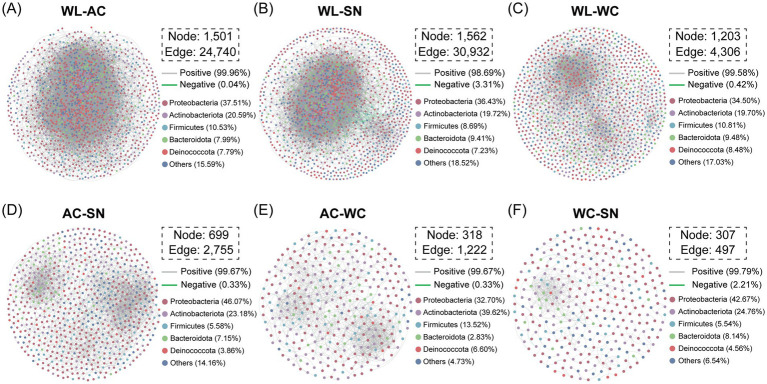
Co-occurrence network analysis of bacterial communities between different dormitory functional areas. Nodes represent individual ASVs, with node size positively correlated with node degree, and colors indicating different modules. Edges represent significant Spearman correlations with *ρ* > 0.6 and *p* < 0.01. Red lines indicate positive correlations, and green lines indicate negative correlations. **(A)** AC-WL, **(B)** WL-SN, **(C)** WC-WL, **(D)** AC-SN, **(E)** AC-WL, **(F)** WC-SN.

Co-occurrence networks were constructed to identify potential microbial interactions within and between different dormitory functional areas. Networks for all samples were established based on Spearman correlation with a threshold of |r| > 0.6 and *p*-value < 0.01 (Hartman et al., 2018; Figure 5; Supplementary Figure S17). A series of key topological features were calculated, including network density, network diameter, average path length and degree (Supplementary Table 5). The entire microbial network consisted of 1,670 nodes linked by 15,808 edges, with all connections being positively correlated (Supplementary Figure S17A), indicating extensive cooperative interactions among microbial taxa. The network, comprising 93 modules, was highly modular with the top five modules accounting for 83.3% of all nodes. Among these major modules, 82.4% of the nodes belonged to the phyla Proteobacteria, Actinobacteriota, Firmicutes, Bacteroidota, and Deinococcota. Actinobacteriota showed the highest presence in all but Module II, whereas other phyla like Patescibacteria and Deinococcota varied in abundance between modules (Supplementary Table 6). Similarly, the pathogen-specific co-occurrence network exhibited a robust interconnected structure, with all connections displaying positive correlations (Supplementary Figure S17B). This network, with fewer nodes, exhibited high modularity (0.444) and a long average path length (APL) of 12, indicating that the indoor pathogen network comprised bacteria with significant functional or ecological niche differences and relatively weak interactions among members. Notably, Proteobacteria remained a major phylum within the top five modules, reflecting its significant role in shaping pathogen community composition (Supplementary Table 7).

Co-occurrence networks for different pairs of functional areas (AC-SN, AC-WC, AC-WL, WC-SN, WL-SN, and WC-WL) were also constructed to explore potential relationships between dormitory functional areas. Across all pairwise co-occurrence networks (Figure 5), nodes were largely associated with five main phyla, namely Proteobacteria, Actinobacteriota, Deinococcota, Firmicutes and Bacteroidota, though the proportions of these phyla varied markedly across different pairwise functional areas. By investigating the topological features within each network, we observed that WL-involved networks (SN-WL, AC-WL, and WC-WL) exhibited higher node and edge counts compared to WL-excluded networks. Furthermore, the WL-SN and AC-WL networks displayed the two highest Average Degree values (39.606 and 32.965, respectively) and the two lowest Average Path Lengths (3.354 and 3.585, respectively). These metrics indicated a more compact and tightly linked relationship among microbial communities composed of floor microbes compared to other non-WL networks. Overall, the WL-involved networks facilitated a more integrated and dense microbial community, demonstrating that floor-associated microbes possess greater connectivity and cohesion than those found in other dormitory functional areas.

Source contribution analysis (SCR) was further applied to reveal the connectivity among microbial taxa at functional areas (AC, WC, and SN) and those present on the floor (WL) (Supplementary Figure S18). For bacterial communities, WL was mainly influenced by WC and AC, which accounted for 26.83 and 23.59% of the variation, respectively, whereas SN had a smaller influence with only 5.6%. For pathogens, the influence of all functional areas on WL-associated taxa decreased significantly, with AC dropping to 6.37%, SN to 4.32%, and WC to 4.70%.

### 3.5 Assembly process of bacterial community in the dormitory

**Figure 6:**
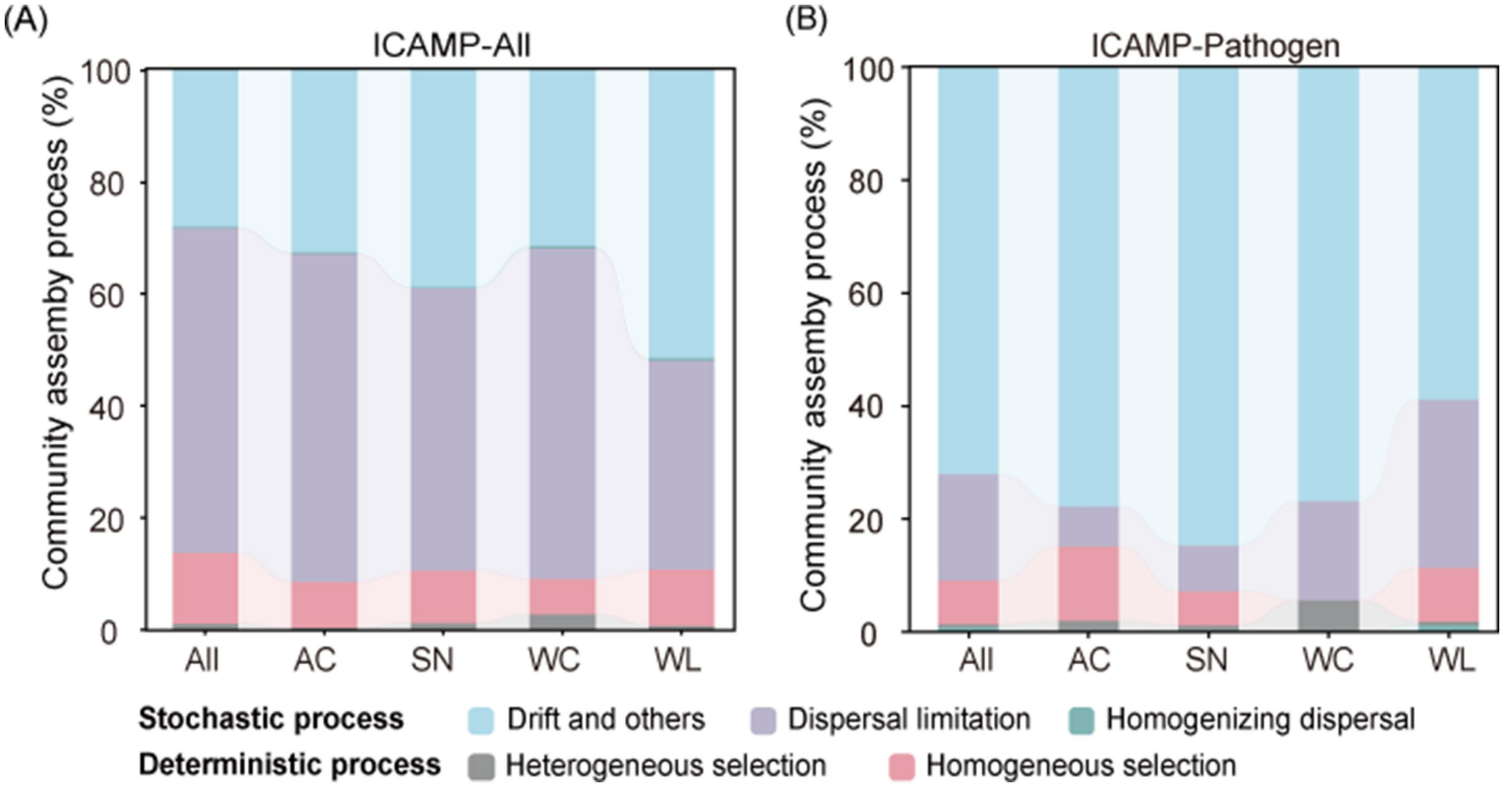
The relative contribution of different processes to the assembly of **(A)** the overall bacterial taxa and **(B)** potential pathogen communities.

To further elucidate the mechanisms driving the microbial community assembly, iCAMP, an approach that includes null models as its methodology to distinguish between different community assembly processes, was employed. Stochastic processes significantly influenced the assembly of bacterial community in the dormitory (86.28%), with dispersal limitation being the dominant process (58.05%) (Figure 6A). Deterministic processes also mattered in shaping the microbial communities (13.72%), where homogeneous selection played a pivotal role (12.64%). Although the relative influence of assembly processes varied spatially within the dormitory, stochastic processes consistently contributed a significant portion of the community assembly. Specifically, AC, SN, and WC were primarily influenced by dispersal limitation (contributed 58.75, 50.55 and 59.00%, respectively), whereas diversification drift was more pronounced in the WL samples (51.43%). The same stochastic-process-dominating pattern was observed in the bacterial pathogens (91.72%) (Figure 6B), with drift being the predominant stochastic process explaining the pathogen community variation (72.16%). Pathogens in WC group exhibited the least influence from deterministic processes, whereas AC samples were more obviously affected, particularly by homogeneous selection. However, in none of the four indoor functional areas did the contribution of deterministic processes exceed 15%. These results reflected that indoor microorganisms and pathogens are more often subjected to stochastic processes, mainly controlled by dispersal limitations and ecological drift.

## 4 Discussion

The crowded and enclosed nature of on-campus dormitories, serving as the primary place for students’ daily activities, has fostered the accumulation and spread of microorganisms and pathogens within this distinct indoor ecosystem, which potentially influence human health. Accordingly, our study not only explored the spatial patterns of indoor microbial communities but also characterized the distributions and diversity of potential pathogenic communities to enhance our understanding of public health risks.

Studies from university dormitories have revealed that indoor environments often harbor unique and diverse microbiomes (Wu et al., 2021). In this study, the highest relative abundances corresponded mainly to the phyla Actinobacteriota and Proteobacteria as well as a subset of bacterial genera such as *Pseudonocardia* and *Kocuria*, which aligns well with previously reported indoor microbial compositions (Ye et al., 2021a; Ruiz-Gil et al., 2020; Madsen et al., 2023; Chudzik et al., 2024). Occupants, by releasing human-associated microorganisms and transporting microbes from outdoor air, serve as a vital source of the indoor microbiome. Therefore, it is not surprising that genera commonly found on human skin and in the respiratory tract, namely, *Staphylococcus* and *Corynebacterium,* were prominently observed in our study (Kolikonda et al., 2017; Fu et al., 2020). Given the typically dense and enclosed nature of university dormitories, an accumulation of diverse human-related microorganisms is likely to occur if not periodically diluted over time (Pausan, 2022). This signifies the necessity of ensuring proper ventilation to facilitate the influx of fresh outdoor air.

Beta-diversity analysis detected little difference in compositions among different dormitory rooms (Supplementary Figure S9), suggesting that the impact of occupant characteristics and activities on microbial communities might be negligible compared to the distinct microenvironmental factors present in different functional areas (Figure 3A). Prior studies have demonstrated that occupant properties and behaviors may significantly shape microbiomes of indoor environments (Cao et al., 2021; Amin et al., 2023; Toyoda et al., 2023). The discrepancy might stem from the relatively fixed activity range, small age gap and uniform living habits among dormitory members, resulting in minimal individual characteristic differences between dormitories compared to other indoor environments with different functions, such as educational facilities and home bedrooms. In contrast, the unique microenvironmental characteristics of functional areas within the dormitory fostered notable variations in microbial community composition and function, warranting the investigation of bacterial community heterogeneity across functional areas.

The microbial diversity patterns varied greatly among the four sampling functional areas, with the abundant microbial phyla on the floor showing good congruence with that of the entire dormitory. The floor environment was demonstrated to act as the repository of indoor microorganisms (Gupta et al., 2019), where the highest alpha diversity was detected (Supplementary Figure S7). The Venn diagram and Lefse analysis further exhibited that there were relatively more unique species and significantly enriched indicator species in the WL group (Figure 3). The high microbial diversity and high proportion of unique species detected on the floor implies that the floor environment may heavily harbor various ubiquitous and endemic bacterial taxa. The potentially pivotal role of floor microbes in indoor microbial communities was further confirmed by network analyses (Figure 5). The WL-involved pairwise co-occurrence networks exhibited higher node and edge counts, along with greater average degree values and shorter average path lengths. These findings collectively suggest that the floor environments harbor highly complex and interconnected microbial communities, demonstrating the central role of floor microbial taxa and their transmission relationships with microbes in other functional areas. This was further supported by the results of source contribution analysis, which calculated the influence and migration of microbial communities from functional areas in dormitory rooms to floor microbial taxa. The analysis indicates that the toilet and air conditioning are non-negligible interconnection of floor microbial community in dormitory rooms (Supplementary Figure S18).

The species turnover component explained over 90% of bacterial community dissimilarity (Figures 4A,B), consistent with its dominance across diverse ecosystems (Beca et al., 2017; Soininen et al., 2018). This indicates strong compositional complementarity among dormitory functional areas, reflecting distinct microbial assemblages (Arce-Peña et al., 2022). Stochastic processes predominantly governed community assembly (Figure 6), in line with previous research (Bahram et al., 2016; Chen et al., 2019; Zhou et al., 2020), suggesting limited deterministic influence at fine spatial scales (Roguet et al., 2015; Thompson et al., 2017). Notably, even though deterministic processes contributed less overall (~13%), their ecological relevance should not be underestimated. In particular, homogeneous selection in AC samples suggests that specific environmental conditions, such as stable temperature or airflow patterns in air-conditioned areas, may reflect consistent environmental filtering, potentially enriching specific taxa. Among environmental variables, room temperature emerged as the major contributor to indoor microbial composition (Figure 4C; Supplementary Figure S13), possibly explained by its role in modulating physical, chemical and biological processes in ecosystems (Ficklin et al., 2023).

Shifts in bacterial communities can alter ecosystem functions. Therefore, risk assessments of microbial communities, especially those involving pathogenic microbes, are particularly important due to their threats to public health. In this study, a total of 74 potential human-associated bacterial pathogens were detected, accounting for 0.9% of total ASVs. Among these, the HumPN (human-associated potential nosocomial pathogens) group emerged as the most prevalent type, followed by HumPP (human-associated potential pneumonia pathogens). These results indicate the presence of genera that have been linked to clinical infections; however, caution is warranted in interpreting health risks solely based on genus-level taxonomy or FAPROTAX predictions, as virulence can vary greatly among strains within the same genus. Notably, several genera, including *Acinetobacter*, *Corynebacterium*, *Prevotella*, and *Moraxella*, ranked among the top 10 most abundant in the inferred pathogenic community. Among these, *Acinetobacter* was the most prevalent genus in the pathogenic community and is frequently encountered in hospital indoor air, where it is associated with various nosocomial infections (Wu et al., 2022; Chawla et al., 2023). In our study, *Acinetobacter* was particularly recovered in air conditioning systems, which play a crucial role in indoor air circulation, suggesting that these units may act as reservoirs or vectors for clinically relevant bacteria.

While functional validation of pathogenicity requires targeted molecular assays, such as qPCR or shotgun metagenomics for virulence gene detection, the observed patterns highlight the need to integrate air conditioning disinfection into public health protocols. The diversity, assemblages, and interactions of these putative pathogenic genera closely mirrored those of the broader microbial community. However, canonical correlation analysis and Mantel tests revealed that room temperature exerted a stronger influence on the pathogen-associated community structure across different functional areas within the indoor environment (Figure 4F; Supplementary Figures S14, S16). This further supports the role of temperature in pathogen survival and proliferation in both natural and built environments (Wu et al., 2019; Hernández-Cabanyero et al., 2020; Gottel et al., 2024; Chen D. V. et al., 2024). Given the sensitivity of pathogens to temperature changes, indoor climate control, particularly temperature regulation and AC system maintenance, should be considered an essential component of strategies to manage microbial exposure and promote indoor environmental health (Song et al., 2022; Carrazana et al., 2023; Raymenants et al., 2023).

This study provides valuable insights into how functional areas impact indoor microbiomes in hot and humid climates. However, several limitations should be considered to enhance the robustness of the findings. First, the relatively small sample size (*n* = 56) and the snapshot design of this study limited the statistical power and generalizability of our findings, particularly regarding the dynamic nature of microbial communities. Given that room temperature emerged as a key environmental determinant influencing microbial distribution, seasonal variations are likely to play a significant role in shaping microbial and pathogen dynamics (Bowd et al., 2022; Solanki et al., 2024). Therefore, future studies incorporating seasonal and long-term variations with larger sample sizes is necessary to provide a more comprehensive view of microbial evolution and reveal potential cyclical patterns in pathogen prevalence. Second, sample sizes were notably uneven across different functional areas, especially for the toilet (WC) group, which contained only five samples. This imbalance, driven by sequencing failures and the absence of toilet facilities in some dormitories, may have compromised the statistical robustness of group comparisons and introduced potential biases in analyses such as ANOVA and LEfSe. To mitigate this issue, we employed non-parametric methods, which are more resilient to unequal group sizes and distributional assumptions. Third, the exclusive reliance on FAPROTAX predictions for pathogen identification introduces some uncertainty in the results. A more integrated approach combining both culture-dependent techniques and advanced bioinformatic tools will be essential for a fuller understanding of pathogenic communities.

## 5 Conclusion

This study provides a comprehensive analysis of the distribution, environmental drivers, and assembly mechanisms of microbial and pathogenic communities across distinct functional areas within university dormitories. The results indicate that dormitory microbiomes are predominantly composed of human-associated taxa, such as *Staphylococcus*, *Kocuria*, and *Corynebacterium*, with significant compositional differences across various functional areas. Notably, the prevalence of *Acinetobacter*, a highly pathogenic genus linked to multiple human infections, raises important public health concerns. Network analysis reveals robust interactions between floor microbial communities and those from other functional areas, underscoring the central role of floor surfaces in fostering connectivity throughout the indoor environment. These findings underscore the critical need for improved ventilation and sanitation practices in university dormitories, with broader implications for public health risk assessment and indoor microbial exposure management.

## Data Availability

The data supporting the findings of this study are publicly available at: https://github.com/HuaiyuCao/dormitory-microbiome. Further inquiries can be directed to the corresponding author.

## Footnotes

^1^ https://github.com/OpenGene/fastp

^2^ https://github.com/marcelm/cutadapt/

^3^ http://gephi.github.io/

## References

[ref1] Adams R. I. Bateman A. C. Bik H. M. Meadow J. F. (2015). Microbiota of the indoor environment: a meta-analysis. Microbiome 3:49. doi: 10.1186/s40168-015-0108-3, PMID: 26459172 PMC4604073

[ref2] Allen J. G. Marr L. C. (2020). Recognizing and controlling airborne transmission of SARS-CoV-2 in indoor environments. Indoor Air 30:12697. doi: 10.1111/ina.12697, PMID: 32557915 PMC7323102

[ref3] Amin H. Šantl-Temkiv T. Cramer C. Finster K. Real F. G. Gislason T.. (2023). Indoor airborne microbiome and endotoxin: meteorological events and occupant characteristics are important determinants. Environ. Sci. Technol. 57, 11750–11766. doi: 10.1021/acs.est.3c01616, PMID: 37523308 PMC10433529

[ref4] Arce-Peña N. P. Arroyo-Rodríguez V. Avila-Cabadilla L. D. Moreno C. E. Andresen E. (2022). Homogenization of terrestrial mammals in fragmented rainforests: the loss of species turnover and its landscape drivers. Ecol. Appl. 32:e02476. doi: 10.1002/eap.2476, PMID: 34653282

[ref5] Bahram M. Kohout P. Anslan S. Harend H. Abarenkov K. Tedersoo L. (2016). Stochastic distribution of small soil eukaryotes resulting from high dispersal and drift in a local environment. ISME J. 10, 885–896. doi: 10.1038/ismej.2015.164, PMID: 26394006 PMC4796928

[ref6] Baselga A. (2010). Partitioning the turnover and nestedness components of beta diversity. Glob. Ecol. Biogeogr. 19, 134–143. doi: 10.1111/j.1466-8238.2009.00490.x

[ref7] Beca G. Vancine M. H. Carvalho C. S. Pedrosa F. Alves R. S. C. Buscariol D.. (2017). High mammal species turnover in forest patches immersed in biofuel plantations. Biol. Conserv. 210, 352–359. doi: 10.1016/j.biocon.2017.02.033

[ref8] Bowd E. J. Banks S. C. Bissett A. May T. W. Lindenmayer D. B. (2022). Disturbance alters the forest soil microbiome. Mol. Ecol. 31, 419–447. doi: 10.1111/mec.16242, PMID: 34687569

[ref9] Cao L. Yang L. Swanson C. S. Li S. He Q. (2021). Comparative analysis of impact of human occupancy on indoor microbiomes. Front. Environ. Sci. Eng. 15:89. doi: 10.1007/s11783-020-1383-1, PMID: 33425458 PMC7783699

[ref10] Caporaso J. G. Lauber C. L. Walters W. A. Berg-Lyons D. Lozupone C. A. Turnbaugh P. J.. (2011). Global patterns of 16S rRNA diversity at a depth of millions of sequences per sample. Proc. Natl. Acad. Sci. U. S. A. 108, 4516–4522. doi: 10.1073/pnas.1000080107, PMID: 20534432 PMC3063599

[ref11] Carrazana E. Ruiz-Gil T. Fujiyoshi S. Tanaka D. Noda J. Maruyama F.. (2023). Potential airborne human pathogens: a relevant inhabitant in built environments but not considered in indoor air quality standards. Sci. Total Environ. 901:165879. doi: 10.1016/j.scitotenv.2023.165879, PMID: 37517716

[ref12] Chawla H. Anand P. Garg K. Bhagat N. Varmani S. G. Bansal T.. (2023). A comprehensive review of microbial contamination in the indoor environment: sources, sampling, health risks, and mitigation strategies. Front. Public Health 11:1285393. doi: 10.3389/fpubh.2023.1285393, PMID: 38074709 PMC10701447

[ref13] Chen Y. Liang Z. Li G. An T. (2024). Indoor/outdoor airborne microbiome characteristics in residential areas across four seasons and its indoor purification. Environ. Int. 190:108857. doi: 10.1016/j.envint.2024.108857, PMID: 38954924

[ref14] Chen W. Ren K. Isabwe A. Chen H. Liu M. Yang J. (2019). Stochastic processes shape microeukaryotic community assembly in a subtropical river across wet and dry seasons. Microbiome 7:138. doi: 10.1186/s40168-019-0749-8, PMID: 31640783 PMC6806580

[ref15] Chen D. V. Slowinski S. P. Kido A. K. Bruns E. L. (2024). High temperatures reduce growth, infection, and transmission of a naturally occurring fungal plant pathogen. Ecology 105:e4373. doi: 10.1002/ecy.4373 38923499

[ref16] Chudzik A. Jalkanen K. Täubel M. Szponar B. Paściak M. (2024). Identification of environmental Actinobacteria in buildings by means of chemotaxonomy, 16S rRNA sequencing, and MALDI-TOF MS. Microbiol. Spectr. 12:e0359623. doi: 10.1128/spectrum.03596-23, PMID: 38299830 PMC10913483

[ref17] Dai T. Wen D. Bates C. T. Wu L. Guo X. Liu S.. (2022). Nutrient supply controls the linkage between species abundance and ecological interactions in marine bacterial communities. Nat. Commun. 13:175. doi: 10.1038/s41467-021-27857-6, PMID: 35013303 PMC8748817

[ref18] Dunn R. R. Fierer N. Henley J. B. Leff J. W. Menninger H. L. (2013). Home life: factors structuring the bacterial diversity found within and between homes. PLoS One 8:e64133. doi: 10.1371/journal.pone.0064133, PMID: 23717552 PMC3661444

[ref19] Ficklin D. L. Hannah D. M. Wanders N. Dugdale S. J. England J. Klaus J.. (2023). Rethinking river water temperature in a changing, human-dominated world. Nat. Water 1, 125–128. doi: 10.1038/s44221-023-00027-2

[ref20] Fu X. Du B. Meng Y. Li Y. Zhu X. Ou Z.. (2023). Associations between environmental characteristics, high-resolution indoor microbiome, metabolome and allergic and non-allergic rhinitis symptoms for junior high school students. Environ. Sci.: Processes Impacts 25, 791–804. doi: 10.1039/D2EM00480A, PMID: 36883483

[ref21] Fu X. Li Y. Meng Y. Yuan Q. Zhang Z. Norbäck D.. (2020). Associations between respiratory infections and bacterial microbiome in student dormitories in northern China. Indoor Air 30, 816–826. doi: 10.1111/ina.12677, PMID: 32304333

[ref22] Fu X. Li Y. Meng Y. Yuan Q. Zhang Z. Wen H.. (2021). Derived habitats of indoor microbes are associated with asthma symptoms in Chinese university dormitories. Environ. Res. 194:110501. doi: 10.1016/j.envres.2020.110501, PMID: 33221308

[ref23] Gottel N. R. Hill M. S. Neal M. J. Allard S. M. Zengler K. Gilbert J. A. (2024). Biocontrol in built environments to reduce pathogen exposure and infection risk. ISME J. 18:wrad024. doi: 10.1093/ismejo/wrad024, PMID: 38365248 PMC10848226

[ref24] Grydaki N. Colbeck I. Mendes L. Eleftheriadis K. Whitby C. (2021). Bioaerosols in the Athens metro: Metagenetic insights into the PM10 microbiome in a naturally ventilated subway station. Environ. Int. 146:106186. doi: 10.1016/j.envint.2020.106186, PMID: 33126062

[ref25] Guo B. Zhang L. Sun H. Gao M. Yu N. Zhang Q.. (2022). Microbial co-occurrence network topological properties link with reactor parameters and reveal importance of low-abundance genera. NPJ Biofilms Microbiomes 8:3. doi: 10.1038/s41522-021-00263-y, PMID: 35039527 PMC8764041

[ref26] Gupta M. Lee S. Bisesi M. Lee J. (2019). Indoor microbiome and antibiotic resistance on floor surfaces: An exploratory study in three different building types. Int. J. Environ. Res. Public Health 16:4160. doi: 10.3390/ijerph16214160, PMID: 31661921 PMC6862025

[ref27] Hartman K. Van Der Heijden M. G. A. Wittwer R. A. Banerjee S. Walser J.-C. Schlaeppi K. (2018). Cropping practices manipulate abundance patterns of root and soil microbiome members paving the way to smart farming. Microbiome 6:14. doi: 10.1186/s40168-017-0389-9, PMID: 29338764 PMC5771023

[ref28] Hernández-Cabanyero C. Sanjuán E. Fouz B. Pajuelo D. Vallejos-Vidal E. Reyes-López F. E.. (2020). The effect of the environmental temperature on the adaptation to host in the zoonotic pathogen *Vibrio vulnificus*. Front. Microbiol. 11:489. doi: 10.3389/fmicb.2020.00489, PMID: 32296402 PMC7137831

[ref29] Hoisington A. J. Stamper C. E. Bates K. L. Stanislawski M. A. Flux M. C. Postolache T. T.. (2023). Human microbiome transfer in the built environment differs based on occupants, objects, and buildings. Sci. Rep. 13:6446. doi: 10.1038/s41598-023-33719-6, PMID: 37081054 PMC10116103

[ref30] Kim K.-H. Kabir E. Jahan S. A. (2018). Airborne bioaerosols and their impact on human health. J. Environ. Sci. 67, 23–35. doi: 10.1016/j.jes.2017.08.027, PMID: 29778157 PMC7128579

[ref31] Klassert T. E. Leistner R. Zubiria-Barrera C. Stock M. López M. Neubert R.. (2021). Bacterial colonization dynamics and antibiotic resistance gene dissemination in the hospital environment after first patient occupancy: a longitudinal metagenetic study. Microbiome 9:169. doi: 10.1186/s40168-021-01109-7, PMID: 34380550 PMC8359561

[ref32] Klepeis N. E. Nelson W. C. Ott W. R. Robinson J. P. Tsang A. M. Switzer P.. (2001). The National Human Activity Pattern Survey (NHAPS): a resource for assessing exposure to environmental pollutants. J. Expo. Sci. Environ. Epidemiol. 11, 231–252. doi: 10.1038/sj.jea.7500165, PMID: 11477521

[ref33] Kolikonda M. K. Jayakumar P. Sriramula S. Lippmann S. (2017). *Kocuria kristinae* infection during adalimumab treatment. Postgrad. Med. 129, 296–298. doi: 10.1080/00325481.2017.1250606, PMID: 27756166

[ref34] Labouyrie M. Ballabio C. Romero F. Panagos P. Jones A. Schmid M. W.. (2023). Patterns in soil microbial diversity across Europe. Nat. Commun. 14:3311. doi: 10.1038/s41467-023-37937-4, PMID: 37291086 PMC10250377

[ref35] Leung M. H. Y. Tong X. Lee P. K. H. (2019). Indoor microbiome and airborne pathogens. Comprehensive biotechnology 1, 96–106. doi: 10.1016/B978-0-444-64046-8.00477-8

[ref36] Leung M. H. Y. Wilkins D. Li E. K. T. Kong F. K. F. Lee P. K. H. (2014). Indoor-air microbiome in an urban Subway network: diversity and dynamics. Appl. Environ. Microbiol. 80, 6760–6770. doi: 10.1128/AEM.02244-14, PMID: 25172855 PMC4249038

[ref37] Li S. Yang Z. Hu D. Cao L. He Q. (2021). Understanding building-occupant-microbiome interactions toward healthy built environments: a review. Front. Environ. Sci. Eng. 15:65. doi: 10.1007/s11783-020-1357-3, PMID: 33145119 PMC7596174

[ref38] Louca S. Parfrey L. W. Doebeli M. (2016). Decoupling function and taxonomy in the global ocean microbiome. Science 353, 1272–1277. doi: 10.1126/science.aaf4507, PMID: 27634532

[ref39] Madsen A. M. Moslehi-Jenabian S. Frankel M. White J. K. Frederiksen M. W. (2023). Airborne bacterial species in indoor air and association with physical factors. UCL Open Environ. 5:56. doi: 10.14324/111.444/ucloe.000056, PMID: 37229345 PMC10208329

[ref40] Mutsch B. Heiber M. Grätz F. Hain R. Schönfelder M. Kaps S.. (2022). Aerosol particle emission increases exponentially above moderate exercise intensity resulting in superemission during maximal exercise. Proc. Natl. Acad. Sci. USA 119:e2202521119. doi: 10.1073/pnas.2202521119, PMID: 35605123 PMC9295808

[ref41] Oberauner L. Zachow C. Lackner S. Högenauer C. Smolle K.-H. Berg G. (2013). The ignored diversity: complex bacterial communities in intensive care units revealed by 16S pyrosequencing. Sci. Rep. 3:1413. doi: 10.1038/srep01413, PMID: 23475210 PMC3593336

[ref42] Oksanen J. Simpson G. L. Blanchet F. G. Kindt R. Legendre P. (2022). *Vegan: community ecology package. R package version: 2.6–4*

[ref43] Pausan M.-R. (2022). The sanitary indoor environment—a potential source for intact human-associated anaerobes. NPJ Biofilms Microbiomes 8:305. doi: 10.1038/s41522-022-00305-z, PMID: 35650275 PMC9160270

[ref44] Qian J. Hospodsky D. Yamamoto N. Nazaroff W. W. Peccia J. (2012). Size-resolved emission rates of airborne bacteria and fungi in an occupied classroom. Indoor Air 22, 339–351. doi: 10.1111/j.1600-0668.2012.00769.x, PMID: 22257156 PMC3437488

[ref45] Quast C. Pruesse E. Yilmaz P. Gerken J. Schweer T. Yarza P.. (2012). The SILVA ribosomal RNA gene database project: improved data processing and web-based tools. Nucleic Acids Res. 41, D590–D596. doi: 10.1093/nar/gks1219, PMID: 23193283 PMC3531112

[ref46] Raymenants J. Geenen C. Budts L. Thibaut J. Thijssen M. De Mulder H.. (2023). Indoor air surveillance and factors associated with respiratory pathogen detection in community settings in Belgium. Nat. Commun. 14:1332. doi: 10.1038/s41467-023-36986-z, PMID: 36898982 PMC10005919

[ref47] Richardson M. Gottel N. Gilbert J. A. Lax S. (2019). Microbial similarity between students in a common dormitory environment reveals the forensic potential of individual microbial signatures. MBio 10:54. doi: 10.1128/mbio.01054-19 PMC6667619 31363029

[ref48] Roguet A. Laigle G. S. Therial C. Bressy A. Soulignac F. Catherine A.. (2015). Neutral community model explains the bacterial community assembly in freshwater lakes. FEMS Microbiol. Ecol. 91:fiv125. doi: 10.1093/femsec/fiv125, PMID: 26472576

[ref49] Ruiz-Gil T. Acuña J. J. Fujiyoshi S. Tanaka D. Noda J. Maruyama F.. (2020). Airborne bacterial communities of outdoor environments and their associated influencing factors. Environ. Int. 145:106156. doi: 10.1016/j.envint.2020.106156, PMID: 33039877

[ref50] Sharpe T. McGill G. Dancer S. J. King M.-F. Fletcher L. Noakes C. J. (2020). Influence of ventilation use and occupant behaviour on surface microorganisms in contemporary social housing. Sci. Rep. 10:11841. doi: 10.1038/s41598-020-68809-2, PMID: 32678236 PMC7366681

[ref51] Shenhav L. Thompson M. Joseph T. A. Briscoe L. Furman O. Bogumil D.. (2019). FEAST: fast expectation-maximization for microbial source tracking. Nat. Methods 16, 627–632. doi: 10.1038/s41592-019-0431-x, PMID: 31182859 PMC8535041

[ref52] Soininen J. Heino J. Wang J. (2018). A meta-analysis of nestedness and turnover components of beta diversity across organisms and ecosystems. Glob. Ecol. Biogeogr. 27, 96–109. doi: 10.1111/geb.12660

[ref53] Solanki A. C. Gurjar N. S. Sharma S. Wang Z. Kumar A. Solanki M. K.. (2024). Decoding seasonal changes: soil parameters and microbial communities in tropical dry deciduous forests. Front. Microbiol. 15:1258934. doi: 10.3389/fmicb.2024.1258934, PMID: 38440136 PMC10910104

[ref54] Song L. Zhou J. Wang C. Meng G. Li Y. Jarin M.. (2022). Airborne pathogenic microorganisms and air cleaning technology development: a review. J. Hazard. Mater. 424:127429. doi: 10.1016/j.jhazmat.2021.127429, PMID: 34688006

[ref55] Stegen J. C. Lin X. Fredrickson J. K. Chen X. Kennedy D. W. Murray C. J.. (2013). Quantifying community assembly processes and identifying features that impose them. ISME J. 7, 2069–2079. doi: 10.1038/ismej.2013.93, PMID: 23739053 PMC3806266

[ref56] Sun Y. Meng Y. Ou Z. Li Y. Zhang M. Chen Y.. (2022a). Indoor microbiome, air pollutants and asthma, rhinitis and eczema in preschool children – a repeated cross-sectional study. Environ. Int. 161:107137. doi: 10.1016/j.envint.2022.107137, PMID: 35168186

[ref57] Sun Y. Zhang M. Ou Z. Meng Y. Chen Y. Lin R.. (2022b). Indoor microbiome, microbial and plant metabolites, chemical compounds, and asthma symptoms in junior high school students: a multicentre association study in Malaysia. Eur. Respir. J. 60:2200260. doi: 10.1183/13993003.00260-2022, PMID: 35618276 PMC9647074

[ref58] Thompson L. R. Williams G. J. Haroon M. F. Shibl A. Larsen P. Shorenstein J.. (2017). Metagenomic covariation along densely sampled environmental gradients in the Red Sea. ISME J. 11, 138–151. doi: 10.1038/ismej.2016.99, PMID: 27420030 PMC5315489

[ref59] Toyoda A. Shibata Y. Matsuo Y. Terada K. Sugimoto H. Higashi K.. (2023). Diversity and compositional differences of the airborne microbiome in a biophilic indoor environment. Sci. Rep. 13:8179. doi: 10.1038/s41598-023-34928-9, PMID: 37210416 PMC10199911

[ref60] Wang J. Liu T. Sun W. Chen Q. (2020). Bioavailable metal(loid)s and physicochemical features co-mediating microbial communities at combined metal(loid) pollution sites. Chemosphere 260:127619. doi: 10.1016/j.chemosphere.2020.127619, PMID: 32683027

[ref61] Wilkins D. Leung M. H. Lee P. K. (2016). Indoor air bacterial communities in Hong Kong households assemble independently of occupant skin microbiomes. Environ. Microbiol. 18, 1754–1763. doi: 10.1111/1462-2920.12889, PMID: 25923292

[ref62] Wu Z. Lyu H. Liang W. Jing X. Wang Y. Ma X. (2021). Microbial community in indoor dusts from university dormitories: characteristics, potential pathogens and influence factors. Atmos. Pollut. Res. 12, 321–333. doi: 10.1016/j.apr.2020.12.018

[ref63] Wu Z. Lyu H. Ma X. Ren G. Song J. Jing X.. (2022). Comparative effects of environmental factors on bacterial communities in two types of indoor dust: potential risks to university students. Environ. Res. 203:111869. doi: 10.1016/j.envres.2021.111869, PMID: 34411549

[ref64] Wu G. Yang J. Jiang H. Deng Y. Lear G. (2019). Distribution of potentially pathogenic bacteria in the groundwater of the Jianghan plain, Central China. Int. Biodeterior. Biodegrad. 143:104711. doi: 10.1016/j.ibiod.2019.05.028

[ref65] Yang Y. Shi Y. Fang J. Chu H. Adams J. M. (2022). Soil microbial network complexity varies with pH as a continuum, not a threshold, across the North China plain. Front. Microbiol. 13:895687. doi: 10.3389/fmicb.2022.895687, PMID: 35733957 PMC9207804

[ref66] Ye J. Qian H. Zhang J. Sun F. Zhuge Y. Zheng X. (2021a). Combining culturing and 16S rDNA sequencing to reveal seasonal and room variations of household airborne bacteria and correlative environmental factors in Nanjing, Southeast China. Indoor Air 31, 1095–1108. doi: 10.1111/ina.12807, PMID: 33655612

[ref67] Ye J. Qian H. Zhang J. Sun F. Zhuge Y. Zheng X.. (2021b). Concentrations and size-resolved I/O ratios of household airborne bacteria and fungi in Nanjing, Southeast China. Sci. Total Environ. 774:145559. doi: 10.1016/j.scitotenv.2021.145559

[ref68] Young G. R. Sherry A. Smith D. L. (2023). Built environment microbiomes transition from outdoor to human-associated communities after construction and commissioning. Sci. Rep. 13:15854. doi: 10.1038/s41598-023-42427-0, PMID: 37740013 PMC10516947

[ref69] Zhang S. Liang Z. Wang X. Ye Z. Li G. An T. (2023). Bioaerosols in an industrial park and the adjacent houses: dispersal between indoor/outdoor, the impact of air purifier, and health risk reduction. Environ. Int. 172:107778. doi: 10.1016/j.envint.2023.107778, PMID: 36724713

[ref70] Zhou L. Liu L. Chen W.-Y. Sun J.-J. Hou S.-W. Kuang T.-X.. (2020). Stochastic determination of the spatial variation of potentially pathogenic bacteria communities in a large subtropical river. Environ. Pollut. 264:114683. doi: 10.1016/j.envpol.2020.114683, PMID: 32388300

[ref71] Zhou J. Ning D. (2017). Stochastic community assembly: does it matter in microbial ecology? Microbiol. Mol. Biol. Rev. 81, e00002–e00017. doi: 10.1128/MMBR.00002-17, PMID: 29021219 PMC5706748

[ref72] Zhou J.-C. Wang Y.-F. Zhu D. Zhu Y.-G. (2023). Deciphering the distribution of microbial communities and potential pathogens in the household dust. Sci. Total Environ. 872:162250. doi: 10.1016/j.scitotenv.2023.162250, PMID: 36804982

