# Supplementary material for: Functional areas shape indoor microbial structure and potential risks in university dormitories
Source: Front Microbiol. 2025 Jul 15;16:1604064. doi: 10.3389/fmicb.2025.1604064 (PMC12303944; doi:10.3389/fmicb.2025.1604064)
Supplement: Supplementary file 1 [file Data_Sheet_1.docx]

Supplementary Material

# Supplementary Data

**Supplementary Table 1. Contextual Information of Dormitory Rooms and Occupancy Characteristics**

| Dormitory ID | Sampling Date | Floor Level | Number of Residents | Resident Gender | Window Opening Frequency |
| --- | --- | --- | --- | --- | --- |
| jvhanzhai1803 | 20211116 | 18 | 4 | Female | Often |
| qiuse1104 | 20220415 | 11 | 4 | Male | Often |
| qiuse1105 | 20220415 | 11 | 4 | Male | Rarely |
| qiuse1106 | 20220415 | 11 | 4 | Male | Often |
| qiuse1110 | 20220415 | 11 | 4 | Male | Always |
| qiuse1111 | 20220415 | 11 | 4 | Male | Rarely |
| qiuse1118 | 20220415 | 11 | 4 | Male | Always |
| qiuse1119 | 20220415 | 11 | 4 | Male | Often |
| qiuse1124 | 20220415 | 11 | 4 | Male | Always |
| qiuse305 | 20220415 | 3 | 4 | Male | Often |
| qiuse307 | 20220415 | 3 | 4 | Male | Often |
| qiuse315 | 20220415 | 3 | 4 | Male | Always |
| qiuse319 | 20220415 | 3 | 4 | Male | Often |
| qiuse322 | 20220415 | 3 | 4 | Male | Often |
| qiuse323 | 20220415 | 3 | 4 | Male | Often |
| qiuse324 | 20220415 | 3 | 4 | Male | Rarely |
| qiuse326 | 20220415 | 3 | 4 | Male | Often |
| ziweizhai0803 | 20211116 | 8 | 4 | Female | Always |

**Supplementary Table 2. Metadata and Environmental Parameters of Dormitory Samples**

| Dormitory ID | Sample Type | Total Reads | Floor Level | Orientation | Temperature | Relative Humidity | Air Velocity |
| --- | --- | --- | --- | --- | --- | --- | --- |
| jvhanzhai1803 | WL | 292717 | 18 | north | 29.7 | 65.6 | 0.94 |
| jvhanzhai1803 | WC | 353844 | 18 | north | 28.3 | 69 | 2.65 |
| jvhanzhai1803 | AC | 203821 | 18 | north | 29.6 | 65.6 | 0.75 |
| jvhanzhai1803 | SN | 387170 | 18 | north | 27.3 | 72 | 0.99 |
| qiuse1104 | WL | 237369 | 11 | south | 21.9 | 61.3 | 0.58 |
| qiuse1104 | AC | 196798 | 11 | south | 21.9 | 61.8 | 0.99 |
| qiuse1104 | SN | 268388 | 11 | south | 21.7 | 63.4 | 0.68 |
| qiuse1105 | WL | 193738 | 11 | north | 21.4 | 62.7 | 0.57 |
| qiuse1105 | AC | 168512 | 11 | north | 21.6 | 62.2 | 0.75 |
| qiuse1105 | SN | 190957 | 11 | north | 21.4 | 61.9 | 2.05 |
| qiuse1106 | WL | 168016 | 11 | south | 22.1 | 62.2 | 0.52 |
| qiuse1106 | WC | 202072 | 11 | south | 22.6 | 62.2 | 0.66 |
| qiuse1106 | SN | 185173 | 11 | south | 22.1 | 61.7 | 0.57 |
| qiuse1106 | AC | 271882 | 11 | south | 22.3 | 61.8 | 0.61 |
| qiuse1110 | WL | 190696 | 11 | north | 21.9 | 62.2 | 0.66 |
| qiuse1110 | AC | 179200 | 11 | north | 22 | 61.2 | 0.8 |
| qiuse1110 | SN | 153972 | 11 | north | 21.8 | 64 | 0.66 |
| qiuse1111 | WL | 221309 | 11 | north | 21.4 | 61.9 | 0.54 |
| qiuse1111 | AC | 190220 | 11 | north | 21.7 | 62.6 | 0.51 |
| qiuse1111 | SN | 93717 | 11 | north | 21.6 | 61.2 | 0.98 |
| qiuse1118 | WL | 189734 | 11 | south | 22.2 | 61.4 | 0.65 |
| qiuse1118 | AC | 171528 | 11 | south | 22.3 | 61.2 | 0.54 |
| qiuse1118 | SN | 188616 | 11 | south | 22.1 | 61.5 | 0.56 |
| qiuse1119 | WL | 215731 | 11 | north | 21.6 | 68.8 | 0.18 |
| qiuse1119 | AC | 161500 | 11 | north | 21.6 | 68.8 | 0.18 |
| qiuse1119 | SN | 196179 | 11 | north | 21.5 | 64.9 | 0.6 |
| qiuse1124 | WL | 153836 | 11 | north | 21.5 | 65.8 | 0.33 |
| qiuse1124 | WC | 148750 | 11 | north | 21.2 | 68.4 | 0.15 |
| qiuse1124 | AC | 90576 | 11 | north | 21.2 | 66.4 | 0.25 |

**Supplementary Table 2. Metadata and Environmental Parameters of Dormitory Samples** **(Continued 1)**

| Dormitory ID | Sample Type | Total Reads | Floor Level | Orientation | Temperature | Relative Humidity | Air Velocity |
| --- | --- | --- | --- | --- | --- | --- | --- |
| qiuse1124 | SN | 184414 | 11 | north | 22.1 | 63.4 | 0.44 |
| qiuse305 | WL | 294473 | 3 | north | 21.7 | 64.5 | 0.52 |
| qiuse305 | AC | 145892 | 3 | north | 21.9 | 63.4 | 0.62 |
| qiuse307 | WL | 145855 | 3 | north | 21.8 | 61.8 | 0.5 |
| qiuse307 | WC | 161041 | 3 | north | 21.5 | 63.4 | 0.64 |
| qiuse307 | AC | 134677 | 3 | north | 21.9 | 62.1 | 0.5 |
| qiuse307 | SN | 177684 | 3 | north | 21.7 | 61.9 | 0.7 |
| qiuse315 | SN | 204097 | 3 | north | 21.6 | 62.5 | 3.52 |
| qiuse315 | WL | 206025 | 3 | north | 21.7 | 62.2 | 0.62 |
| qiuse319 | WL | 158296 | 3 | south | 21.9 | 63.9 | 0.5 |
| qiuse319 | AC | 143557 | 3 | south | 22.1 | 63.7 | 0.48 |
| qiuse319 | SN | 286317 | 3 | south | 21.9 | 63.6 | 0.57 |
| qiuse322 | WL | 206805 | 3 | south | 22.1 | 63.3 | 0.59 |
| qiuse322 | SN | 224769 | 3 | south | 21.9 | 66.7 | 1.09 |
| qiuse322 | AC | 353029 | 3 | south | 22 | 61 | 1.8 |
| qiuse323 | WL | 275261 | 3 | north | 21.6 | 62.6 | 0.68 |
| qiuse323 | WC | 192957 | 3 | north | 21.5 | 63.6 | 1.08 |
| qiuse323 | AC | 192385 | 3 | north | 21.6 | 63 | 0.47 |
| qiuse323 | SN | 186758 | 3 | north | 21.7 | 61.7 | 1.85 |
| qiuse324 | WL | 204392 | 3 | south | 22 | 64.5 | 0.56 |
| qiuse324 | AC | 240606 | 3 | south | 22.1 | 63 | 0.8 |
| qiuse324 | SN | 248525 | 3 | south | 21.8 | 64.7 | 0.64 |
| qiuse326 | WL | 245702 | 3 | south | 21.7 | 63.3 | 3.54 |
| qiuse326 | AC | 151263 | 3 | south | 21.7 | 65.3 | 0.49 |
| qiuse326 | SN | 240010 | 3 | south | 21.7 | 65 | 1.1 |
| ziweizhai0803 | WL | 307701 | 8 | north | 29.9 | 64.9 | 0.45 |
| ziweizhai0803 | SN | 312709 | 8 | north | 29.7 | 65.2 | 1.05 |

Note: Units are as follows: Temperature: °C; Relative Humidity: %; Air Velocity (Va): m/s.

**Supplementary Table 3.** Average abundance of pathogenic genes.

| **Potential disease group** | **Mean abundance** | **ASV number** |
| --- | --- | --- |
| human nosocomial pathogens (HumPN) | 6.39% | 18 |
| human pneumonia pathogens (HumPP) | 5.12% | 57 |
| human septicemia pathogens (HumPS) | 1.87% | 4 |
| human diarrhea pathogens (HumPD) | 0.01% | 1 |
| human gastroenteritis pathogens (HumPG) | 0.01% | 1 |
| human meningitis pathogens (HumPM) | 0.01% | 1 |

**Supplementary Table 4.** Genus-Level Taxa Showing Statistically Significant Differences Across Functional Areas (*p* < 0.05)

| Genus | Statistic | *p*.value | Method |
| --- | --- | --- | --- |
| *Brevundimonas* | 24.38319 | 2.08E-05 | Kruskal-Wallis |
| *Limnobacter* | 24.27382 | 2.19E-05 | Kruskal-Wallis |
| *Corynebacterium* | 22.14076 | 6.10E-05 | Kruskal-Wallis |
| *Prevotella_9* | 22.03129 | 6.43E-05 | Kruskal-Wallis |
| *Oligella* | 21.51329 | 8.24E-05 | Kruskal-Wallis |
| *Prevotella* | 21.17167 | 9.70E-05 | Kruskal-Wallis |
| *Moraxella* | 19.08632 | 0.000262 | Kruskal-Wallis |
| *Clostridium_sensu_stricto_1* | 17.02746 | 0.000698 | Kruskal-Wallis |
| *uncultured* | 16.98354 | 0.000712 | Kruskal-Wallis |
| *Prevotella_7* | 16.83205 | 0.000765 | Kruskal-Wallis |
| *Dermatophilus* | 16.69046 | 0.000818 | Kruskal-Wallis |
| *Acinetobacter* | 16.05659 | 0.0011 | Kruskal-Wallis |
| *Campylobacter* | 15.57189 | 0.00139 | Kruskal-Wallis |
| *SM2D12* | 15.39351 | 0.00151 | Kruskal-Wallis |
| *Candidatus_Xiphinematobacter* | 13.42755 | 0.0038 | Kruskal-Wallis |
| *Afipia* | 13.39669 | 0.00385 | Kruskal-Wallis |
| *Haemophilus* | 13.0436 | 0.00454 | Kruskal-Wallis |
| *Paenalcaligenes* | 10.99375 | 0.0118 | Kruskal-Wallis |
| *Ottowia* | 10.85985 | 0.0125 | Kruskal-Wallis |
| *Wolbachia* | 10.84016 | 0.0126 | Kruskal-Wallis |
| *Streptococcus* | 10.61024 | 0.014 | Kruskal-Wallis |
| *Lactococcus* | 10.56568 | 0.0143 | Kruskal-Wallis |
| *Serratia* | 10.55879 | 0.0144 | Kruskal-Wallis |
| *Coprococcus* | 10.36521 | 0.0157 | Kruskal-Wallis |
| *Pseudorhodoferax* | 10.35212 | 0.0158 | Kruskal-Wallis |
| *Pusillimonas* | 10.2 | 0.0169 | Kruskal-Wallis |
| *[Eubacterium]_hallii_group* | 10.2 | 0.0169 | Kruskal-Wallis |
| *Lautropia* | 10.01538 | 0.0184 | Kruskal-Wallis |
| *Methylotenera* | 8.580944 | 0.0354 | Kruskal-Wallis |
| *Azohydromonas* | 8.47612 | 0.0371 | Kruskal-Wallis |
| *[Ruminococcus]_torques_group* | 8.077545 | 0.0444 | Kruskal-Wallis |

**Supplementary Table 5.** Topological properties of the co-occurrence network.

| **Topological Parameters** | **All** | **Pathogen** | **AC-SN** | **AC-WC** | **AC-WL** | **SN-WC** | **SN-WL** | **WC-WL** |
| --- | --- | --- | --- | --- | --- | --- | --- | --- |
| Nodes | 1670 | 32 | 699 | 318 | 1501 | 307 | 1562 | 1203 |
| Edges | 15808 | 25 | 2755 | 1222 | 24740 | 497 | 30932 | 4306 |
| Positive correlation | 1 | 100 | 0.9967 | 0.9967 | 0.9996 | 0.9779 | 0.9869 | 0.9958 |
| Negative correlation | 0 | 0 | 0.0033 | 0.0033 | 0.0004 | 0.0221 | 0.0131 | 0.0042 |
| Average Degree (AD) | 18.932 | 1.562 | 7.883 | 7.686 | 32.965 | 3.238 | 39.606 | 7.159 |
| Average Weighted Degree (AWD) | 12.536 | 1.343 | 5.254 | 5.708 | 22.094 | 2.538 | 26.743 | 5.395 |
| Network Diameter (ND) | 19 | 3 | 14 | 14 | 13 | 12 | 16 | 21 |
| Graph Density (GD) | 0.011 | 0.05 | 0.011 | 0.024 | 0.022 | 0.011 | 0.025 | 0.006 |
| Modularity | 0.431 | 0.444 | 0.618 | 0.6 | 0.35 | 0.707 | 0.329 | 0.588 |
| Average Path Length (APL) | 4.703 | 12 | 4.943 | 4.652 | 3.354 | 3.965 | 3.585 | 5.458 |
| Clustering coefficient | 0.317 | 0.412 | 0.279 | 0.359 | 0.303 | 0.327 | 0.367 | 0.252 |
| Module | 93 | 12 | 25 | 12 | 33 | 63 | 60 | 72 |

**Supplementary Table 6.** Phylum-level composition of microorganisms in each module of the co-occurrence network for overall bacterial community.

| **Phylum** | **Module 1** | **Module 2** | **Module 3** | **Module 4** | **Module 5** |
| --- | --- | --- | --- | --- | --- |
| Proteobacteria | 20.562 | 39.370 | 11.062 | 23.156 | 21.647 |
| Actinobacteriota | 49.615 | 17.407 | 63.801 | 57.076 | 34.333 |
| Patescibacteria | 0.115 | 13.148 | 1.172 | 2.353 | 3.725 |
| Deinococcota | 8.615 | 10.037 | 13.357 | 0.143 | 28.292 |
| Firmicutes | 17.769 | 9.074 | 3.450 | 0 | 0.070 |
| Bacteroidota | 0.615 | 5.370 | 4.381 | 4.884 | 7.652 |
| Others | 2.710 | 5.600 | 2.780 | 12.390 | 4.280 |

**Supplementary Table 7.** Phylum-level composition of microorganisms in each module of the co-occurrence network for pathogen communities.

| **Phylum** | **Module 1** | **Module 2** | **Module 3** | **Module 4** | **Module 5** |
| --- | --- | --- | --- | --- | --- |
| Proteobacteria | 0.532 | 100 | 100 | 100 | 100 |
| Actinobacteriota | 0.468 | 0 | 0 | 0 | 0 |


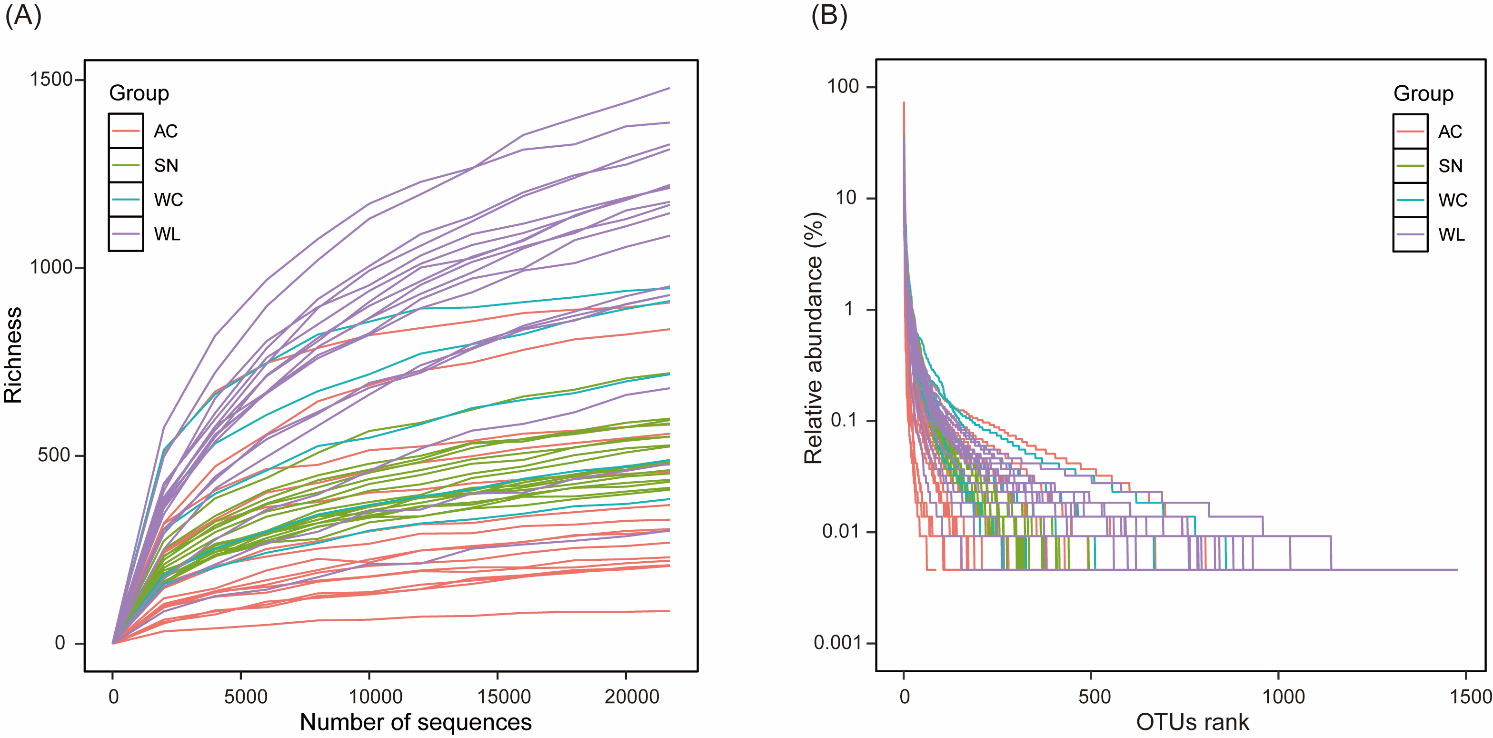


**Supplementary Figure S1.** Assessment of sequencing depth and microbial community structure. (**A**) Rarefaction curves of all samples based on observed ASV richness. (**B**) Rank abundance curves showing the relative abundance of ASVs against their rank in each sample.


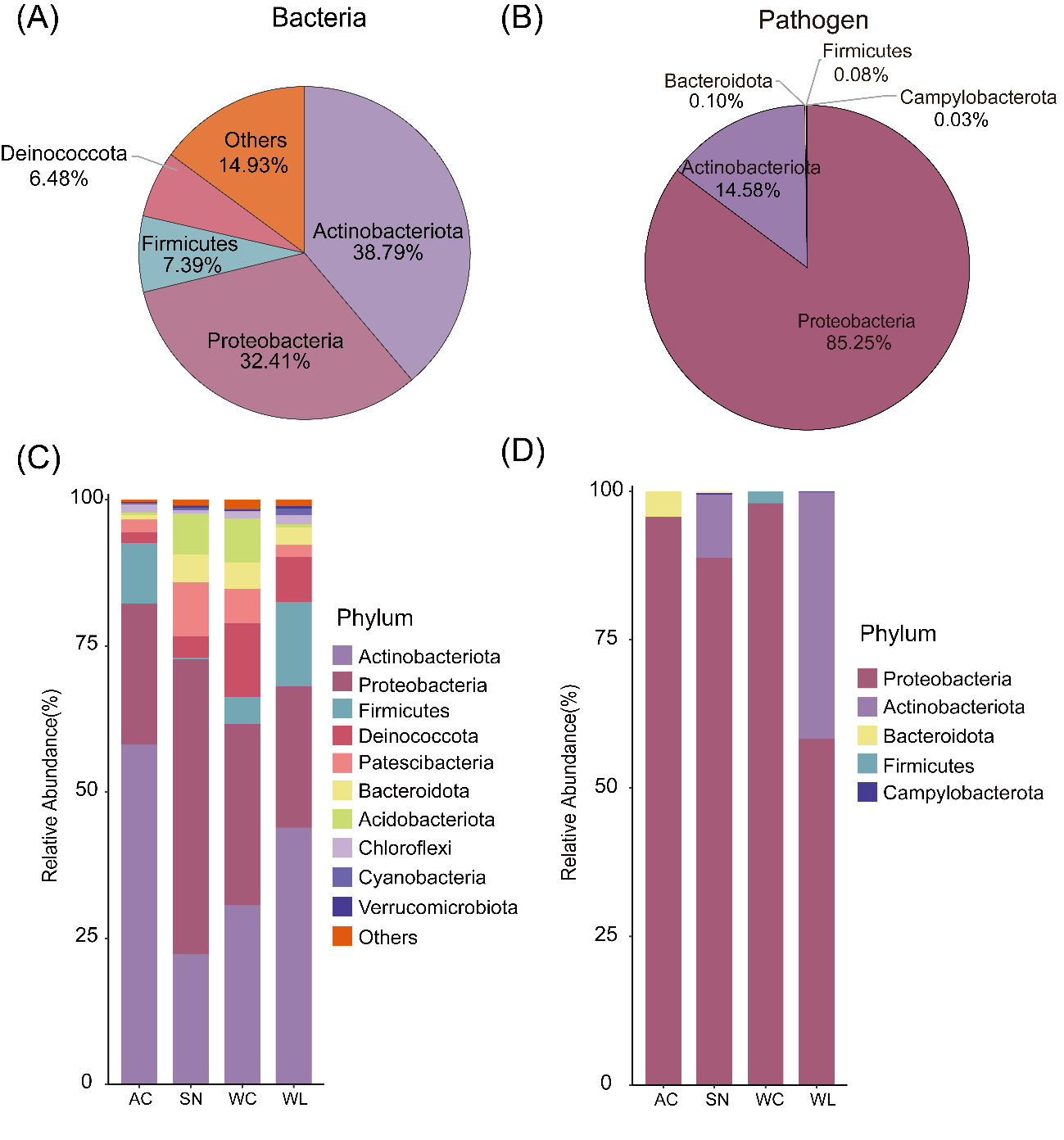


**Supplementary Figure S2.** Microbial species composition in the dormitory. **(A)** Composition of all bacteria at the phylum level; **(B)** Composition of pathogens at the phylum level; **(C)** Composition of the overall bacterial community at the phylum level across functional areas; **(D)** Composition of pathogen communities at the phylum level across functional areas.


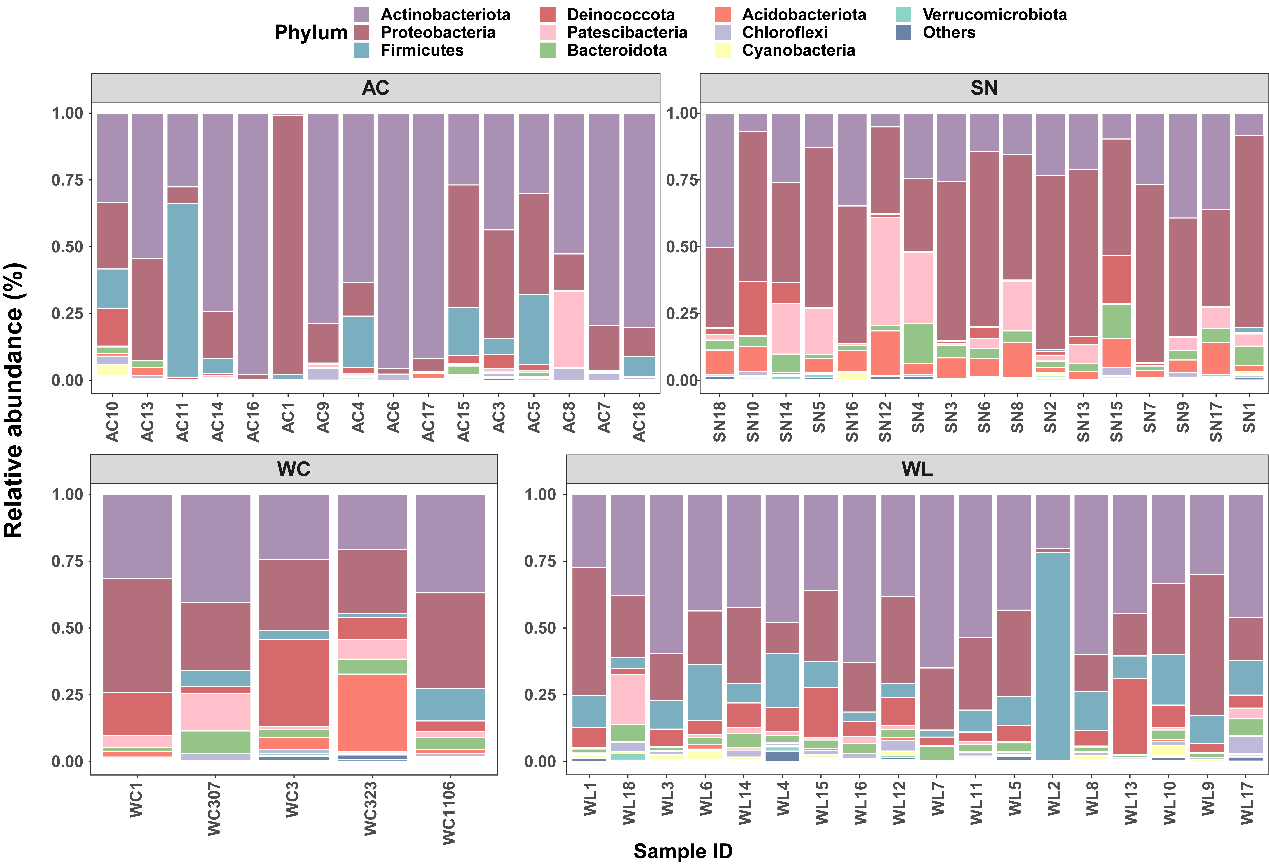


**Supplementary Figure S3.** Composition of bacterial communities at the phylum level in samples from different functional areas.


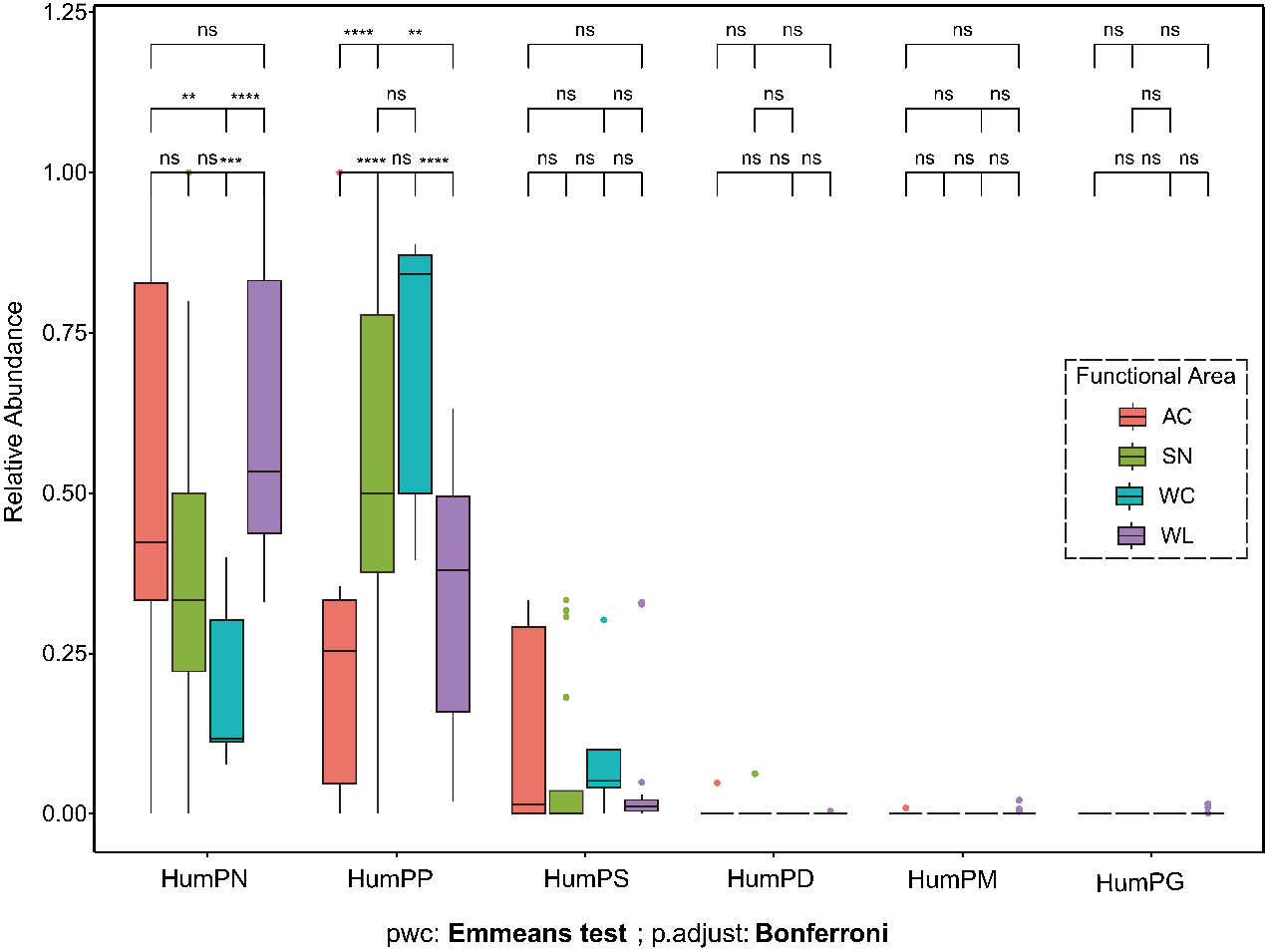


**Supplementary Figure S4.** Relative abundance of pathogenic gene taxa in different groups, with statistical analysis performed using the Emmeans test.


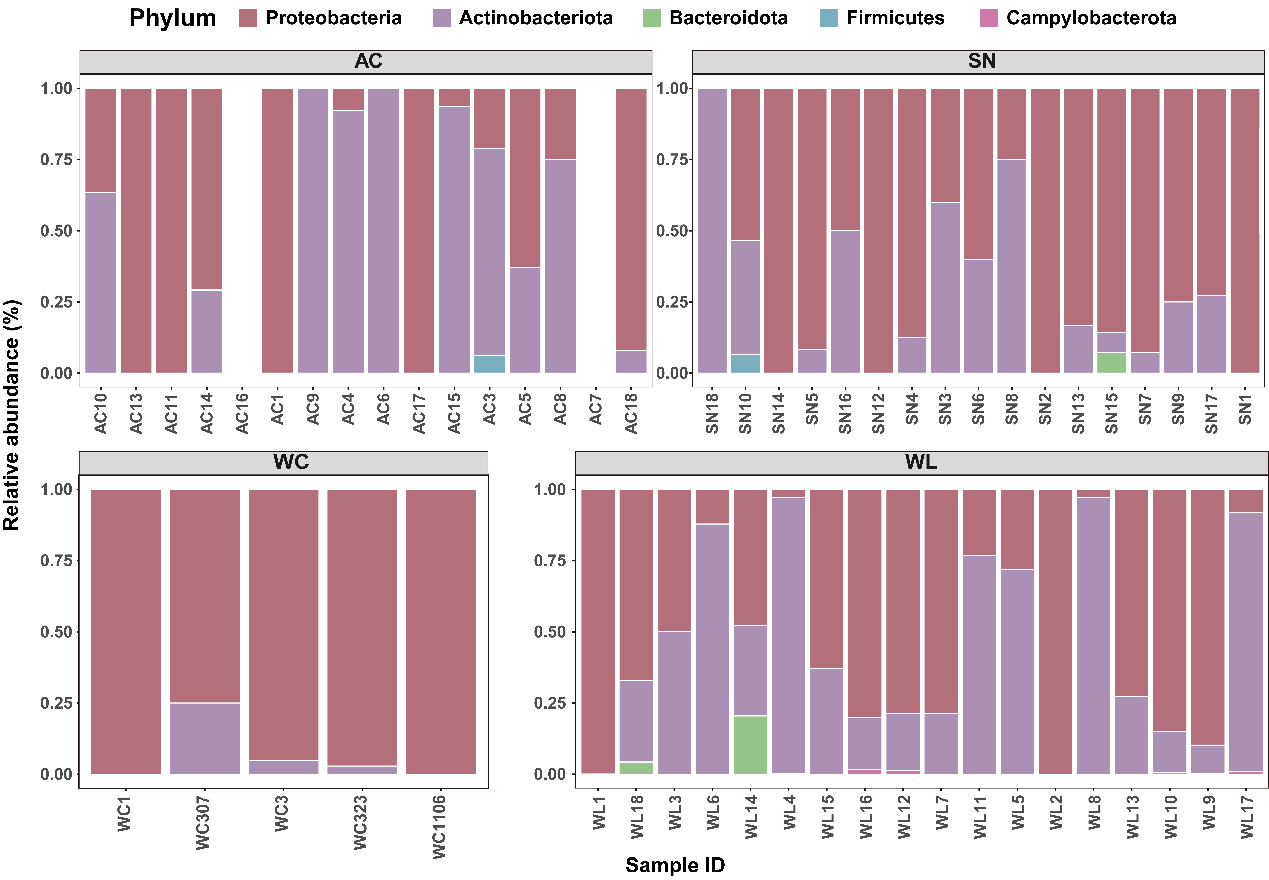


**Supplementary Figure S5.** Composition of pathogen communities at the phylum level in samples from different functional areas.


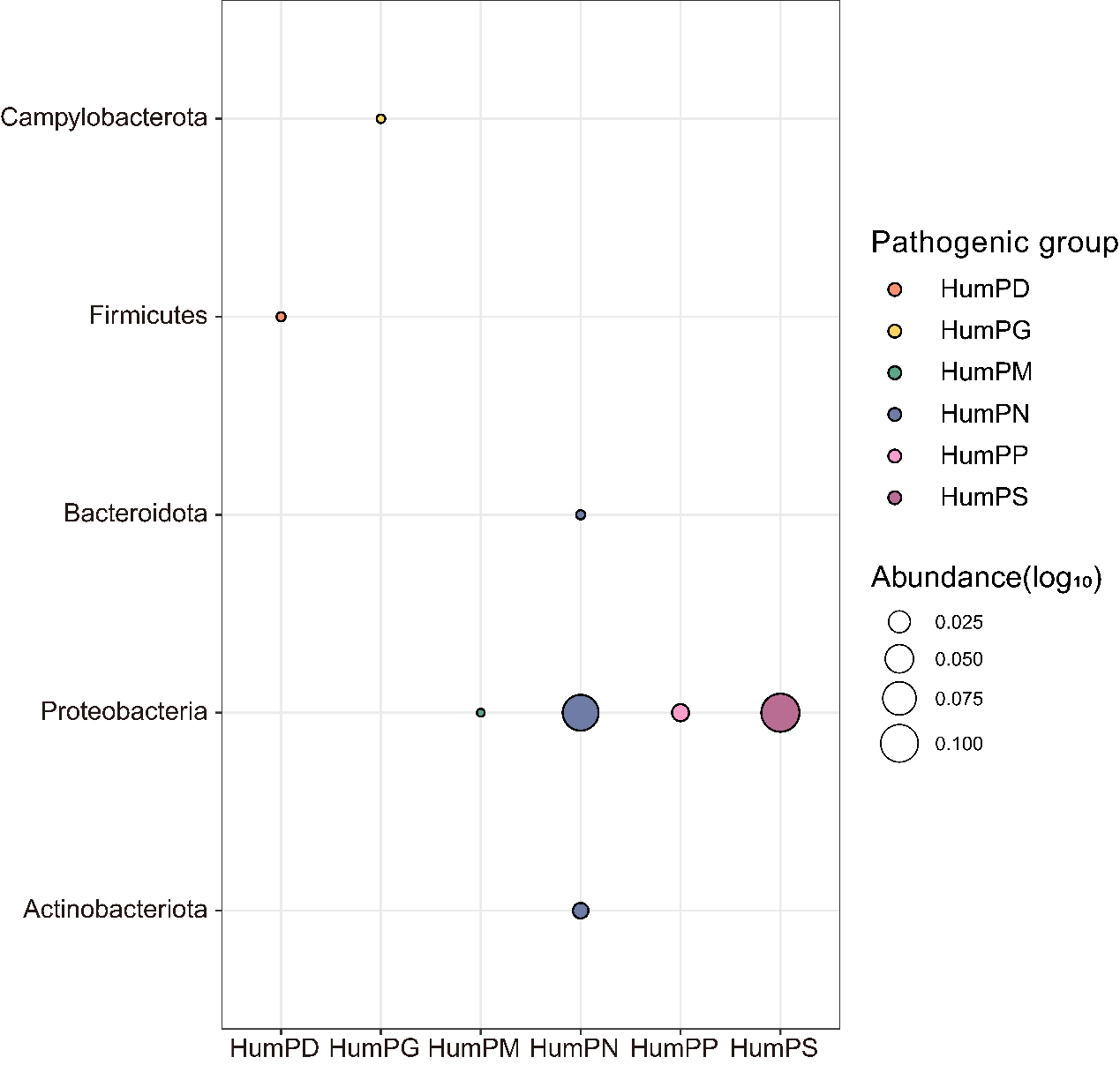


**Supplementary Figure S6.** Relationships between potentially pathogenic groups and taxonomic taxa at the phylum level. Circle size represents abundance; colors indicate different taxa. Values are ln(x + 1)-transformed abundance.


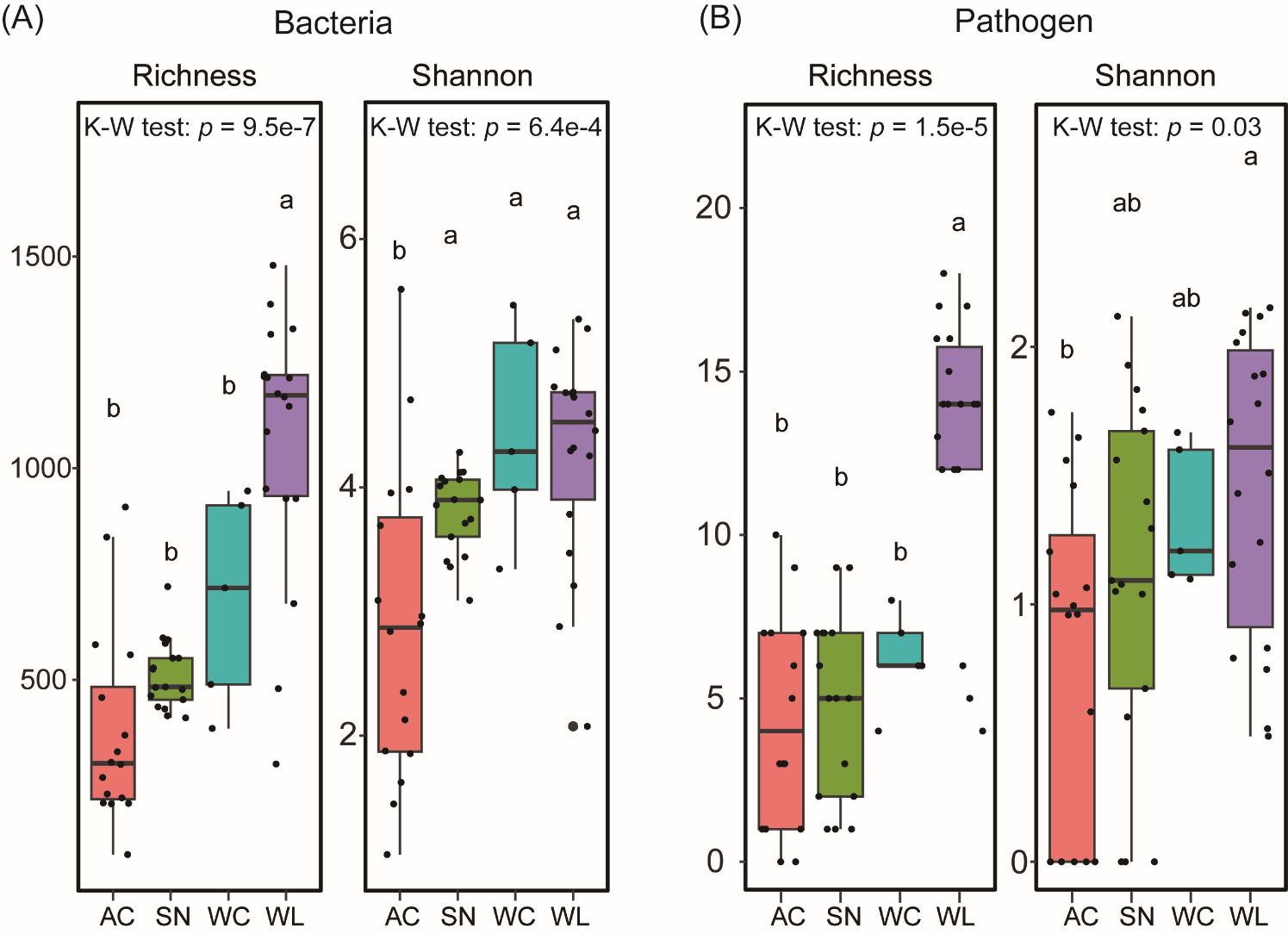


**Supplementary Figure S7.** Alpha diversity of **(A)** the overall bacterial community and **(B)** potential pathogen communities across functional areas. Statistical differences among groups were assessed using the non-parametric Kruskal–Wallis test, followed by Dunn’s post hoc test with Bonferroni correction for multiple comparisons.


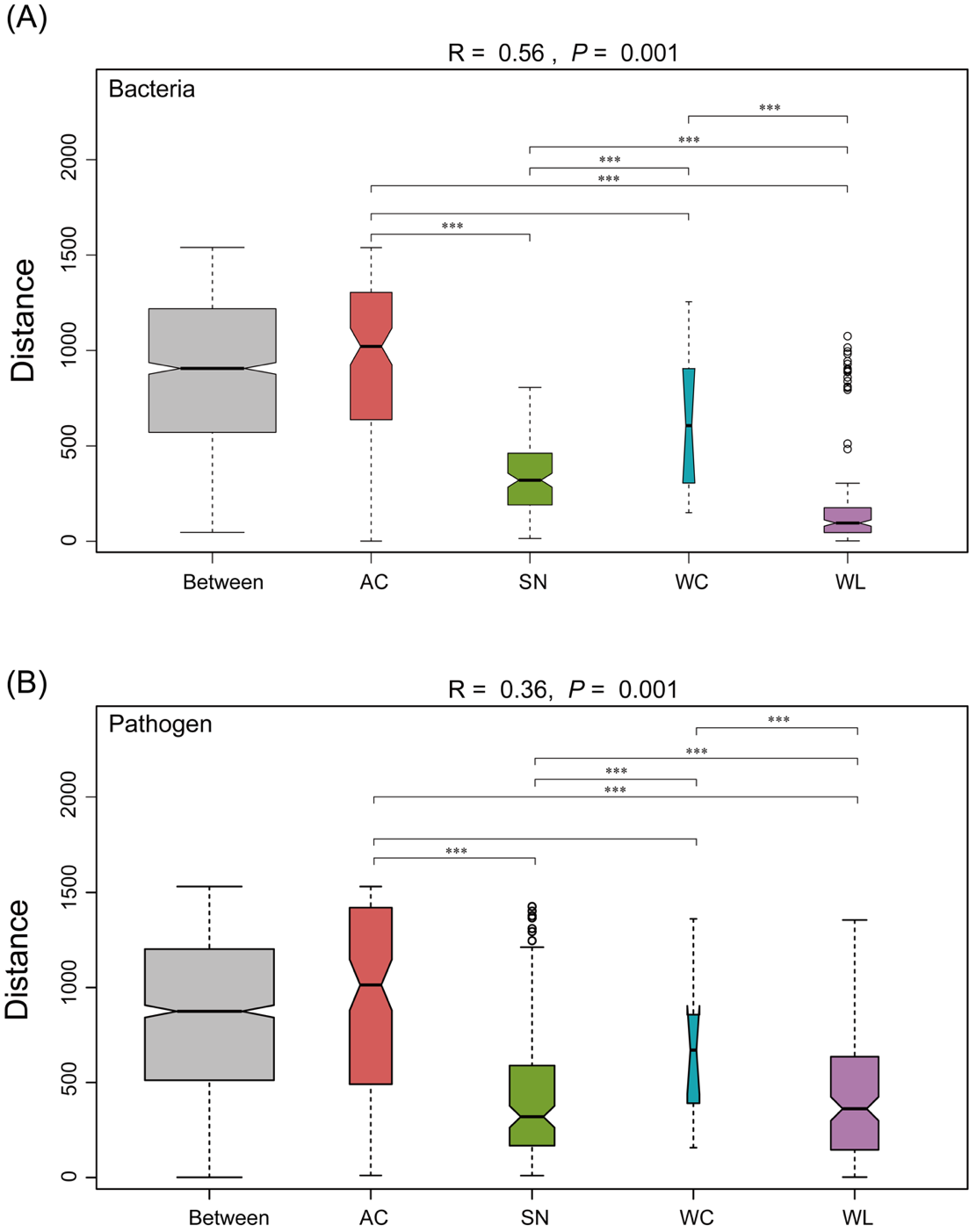


**Supplementary Figure S8.** Beta diversity across functional areas based on ANOSIM results for **(A)** the overall bacterial community and **(B)** potential pathogen communities.


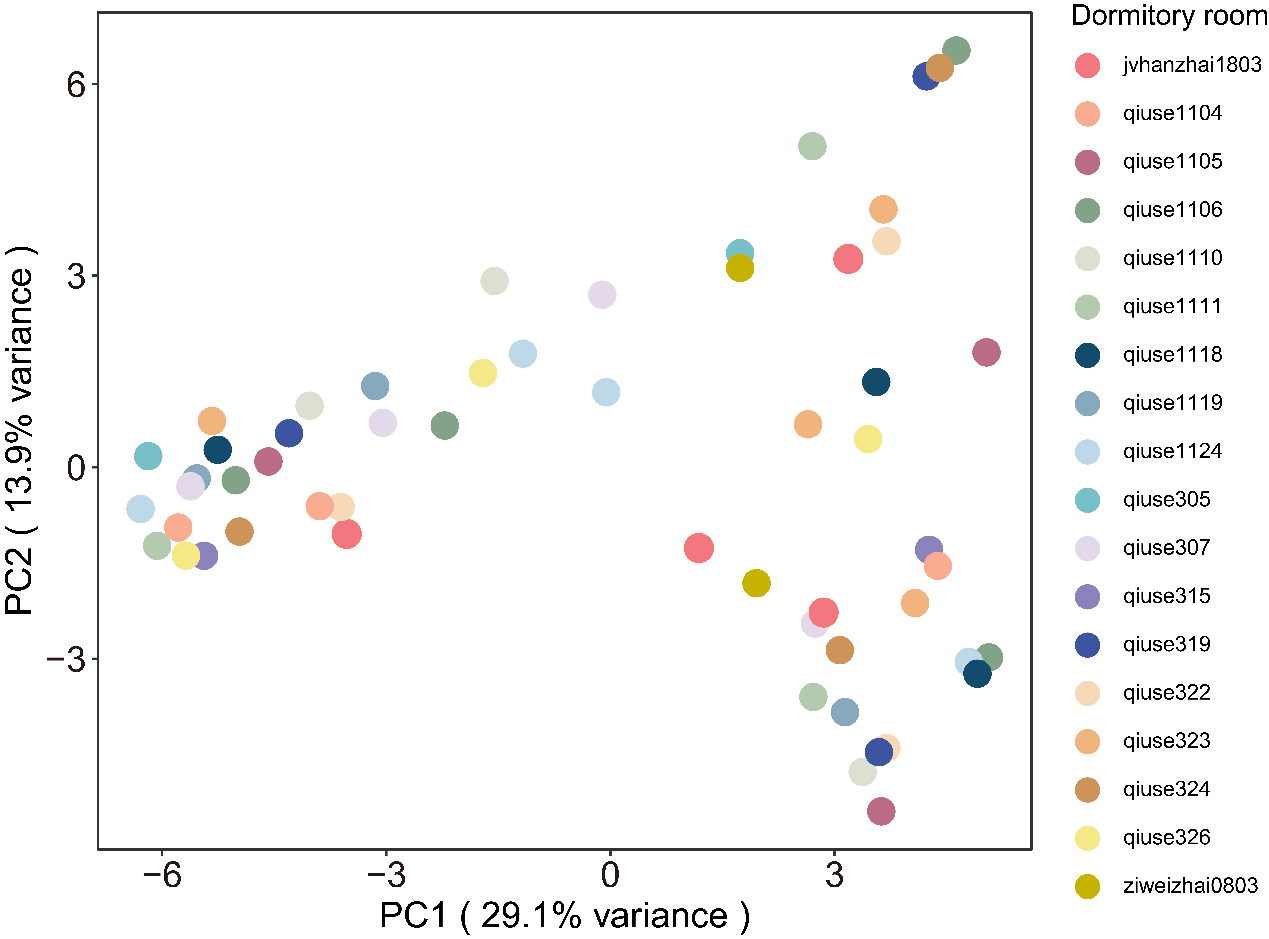


**Supplementary Figure S9.** PCoA of dormitory bacterial communities colored by sampling locations.


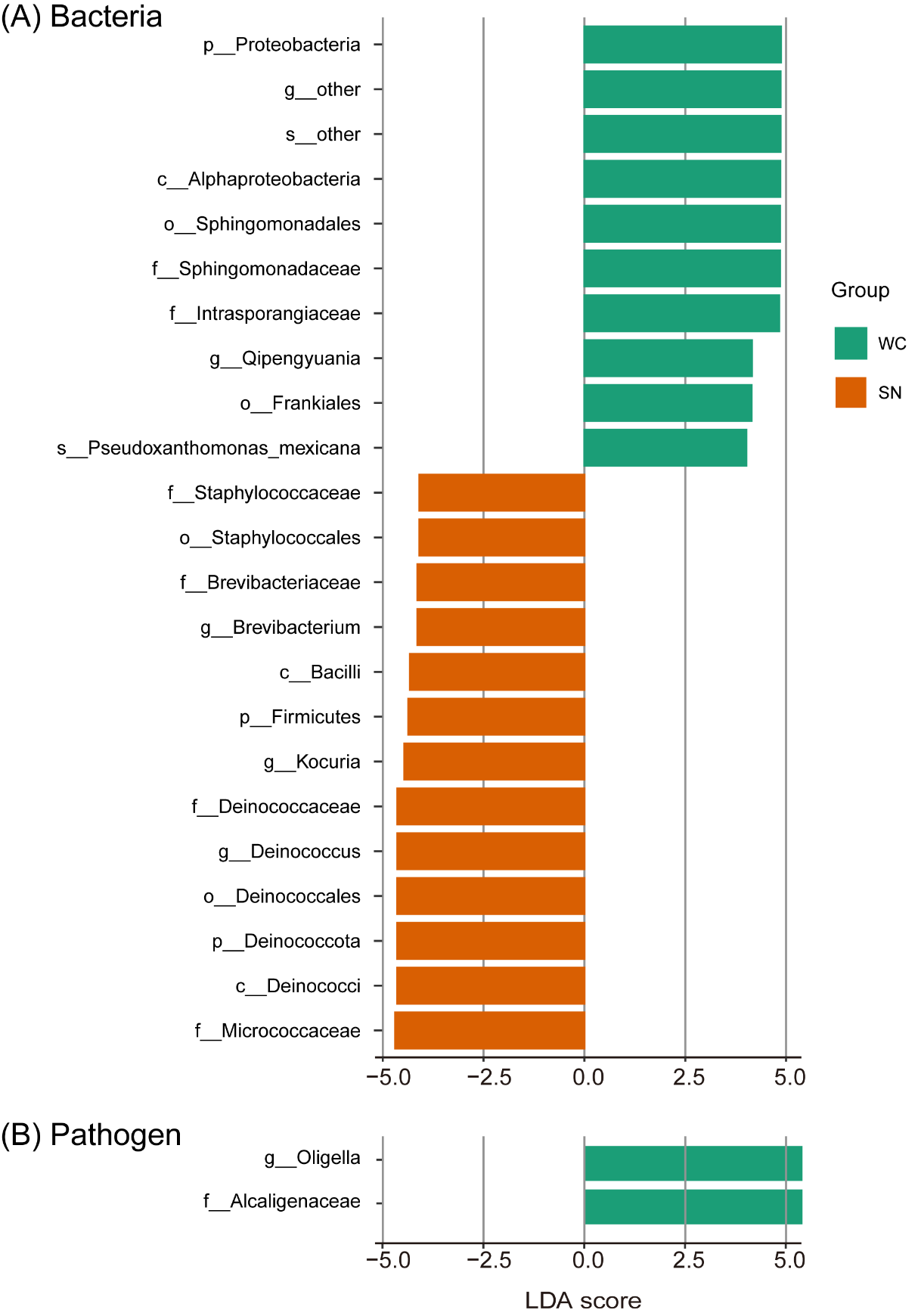


**Supplementary Figure S10.** LDA score plots for the subsampling-based LEfSe analysis of (A) the overall bacterial community and (B) potential pathogen communities in SN and WC groups.


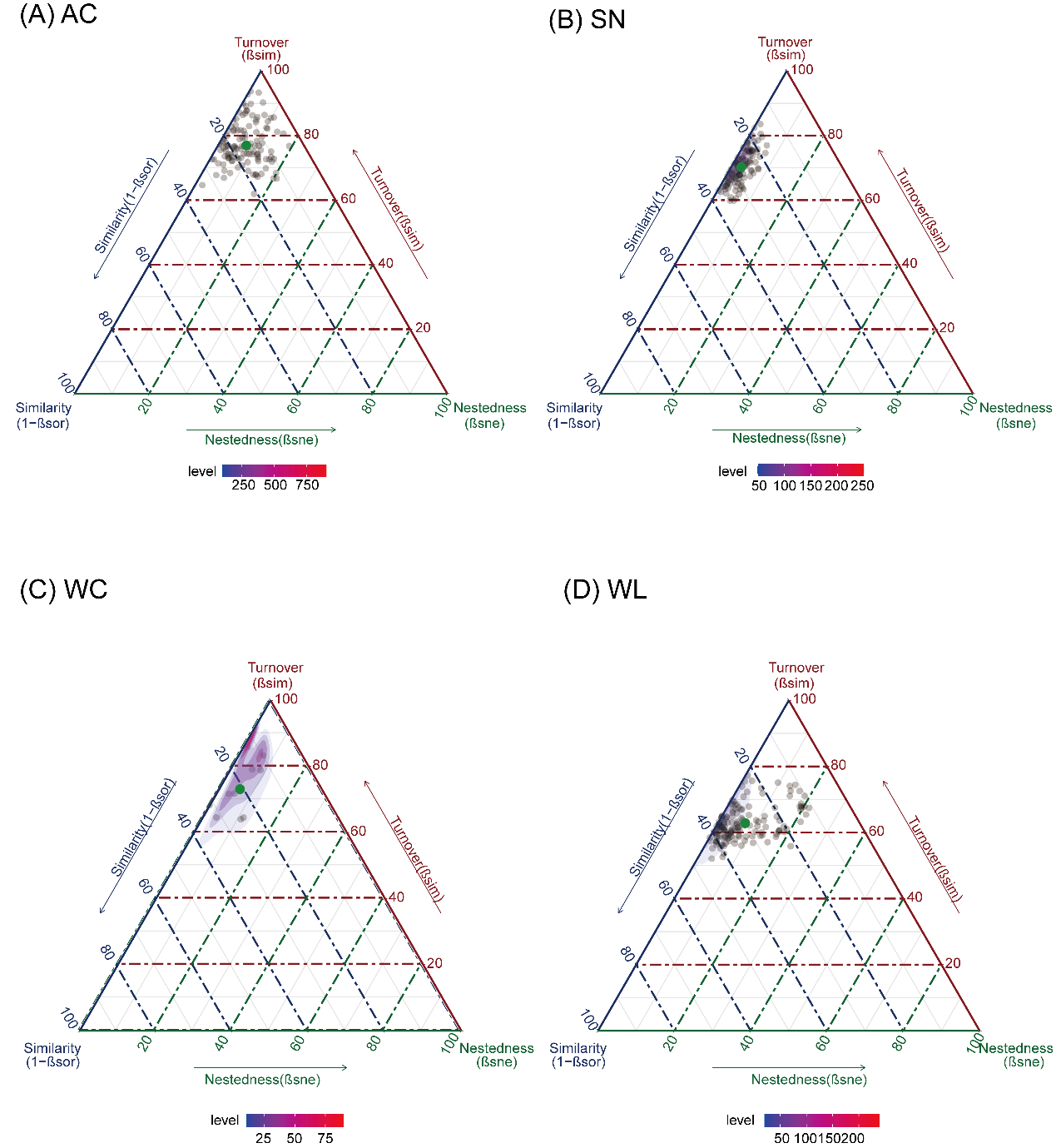


**Supplementary Figure S11.** Ternary plots showing beta diversity partitioning of the overall bacterial community across functional areas: AC **(A)**, SN **(B)**, WC **(C)**, and WL **(D)**.


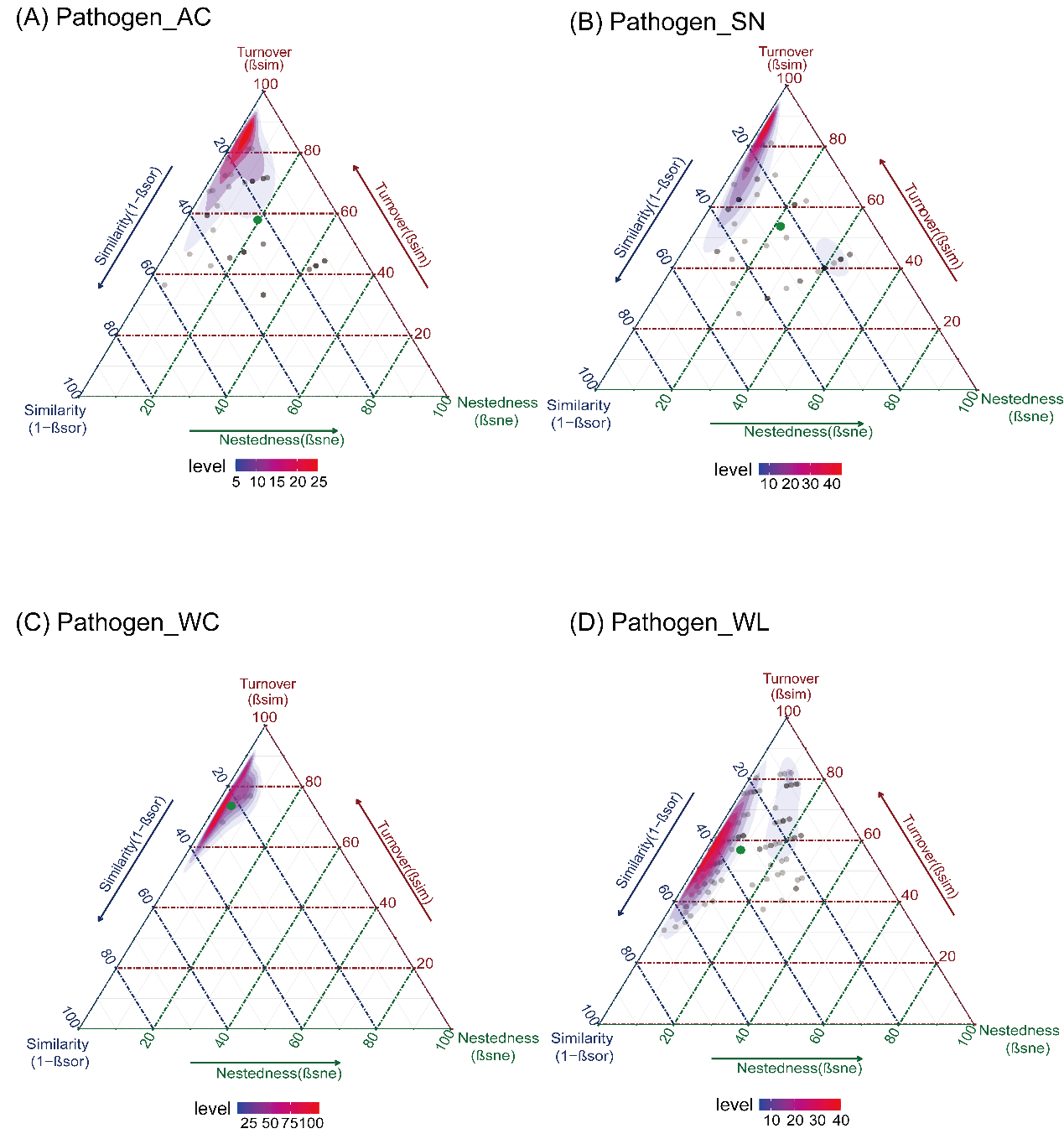


**Supplementary Figure S12.** Ternary plots showing beta diversity partitioning of pathogen communities across functional areas. **(A)** AC, **(B)** SN, **(C)** WC, **(D)** WL.


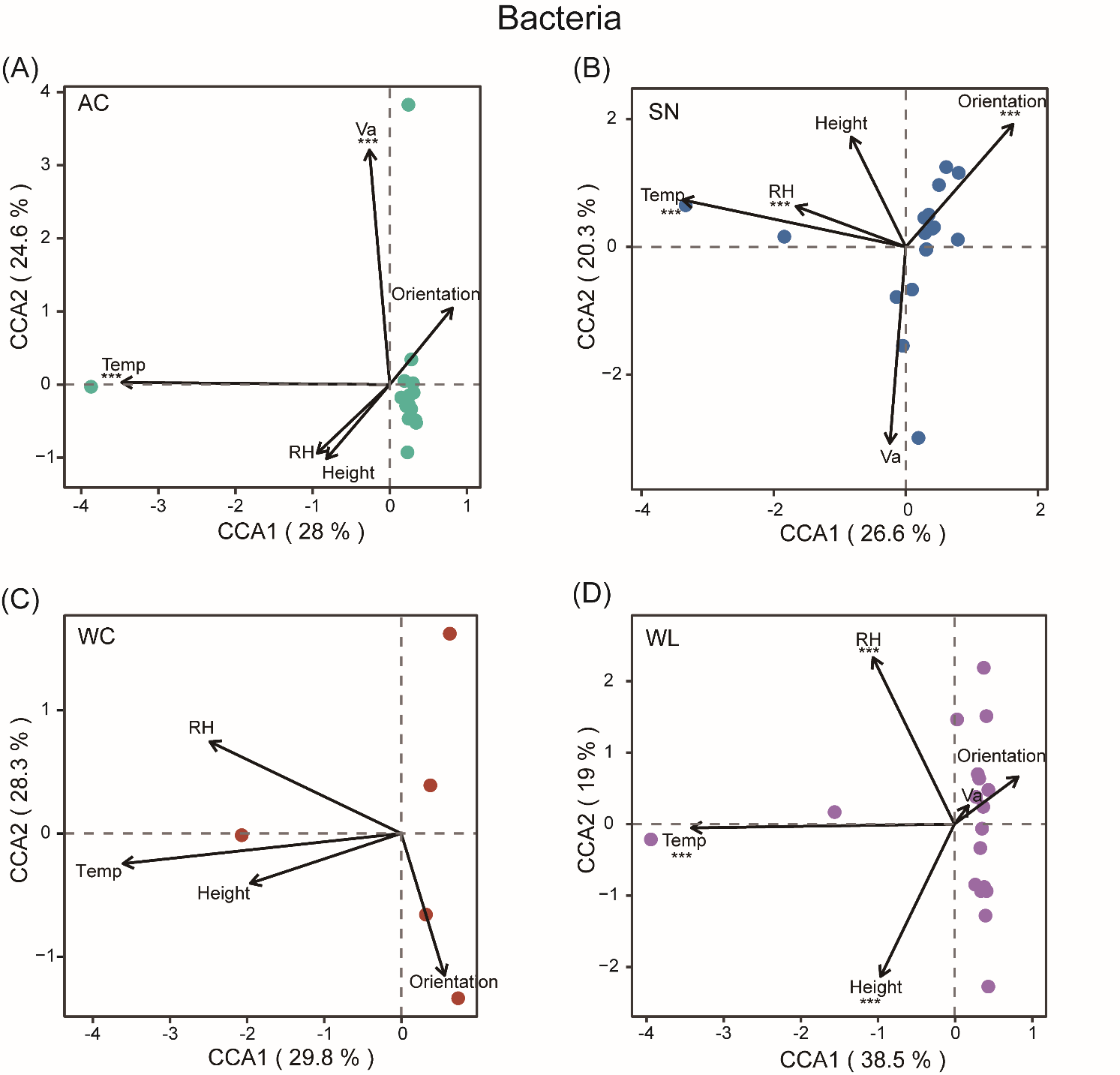


**Supplementary Figure S13.** Canonical correspondence analysis (CCA) of the overall bacterial community across functional areas **(A)** AC, **(B)** SN, **(C)** WC, **(D)** WL.


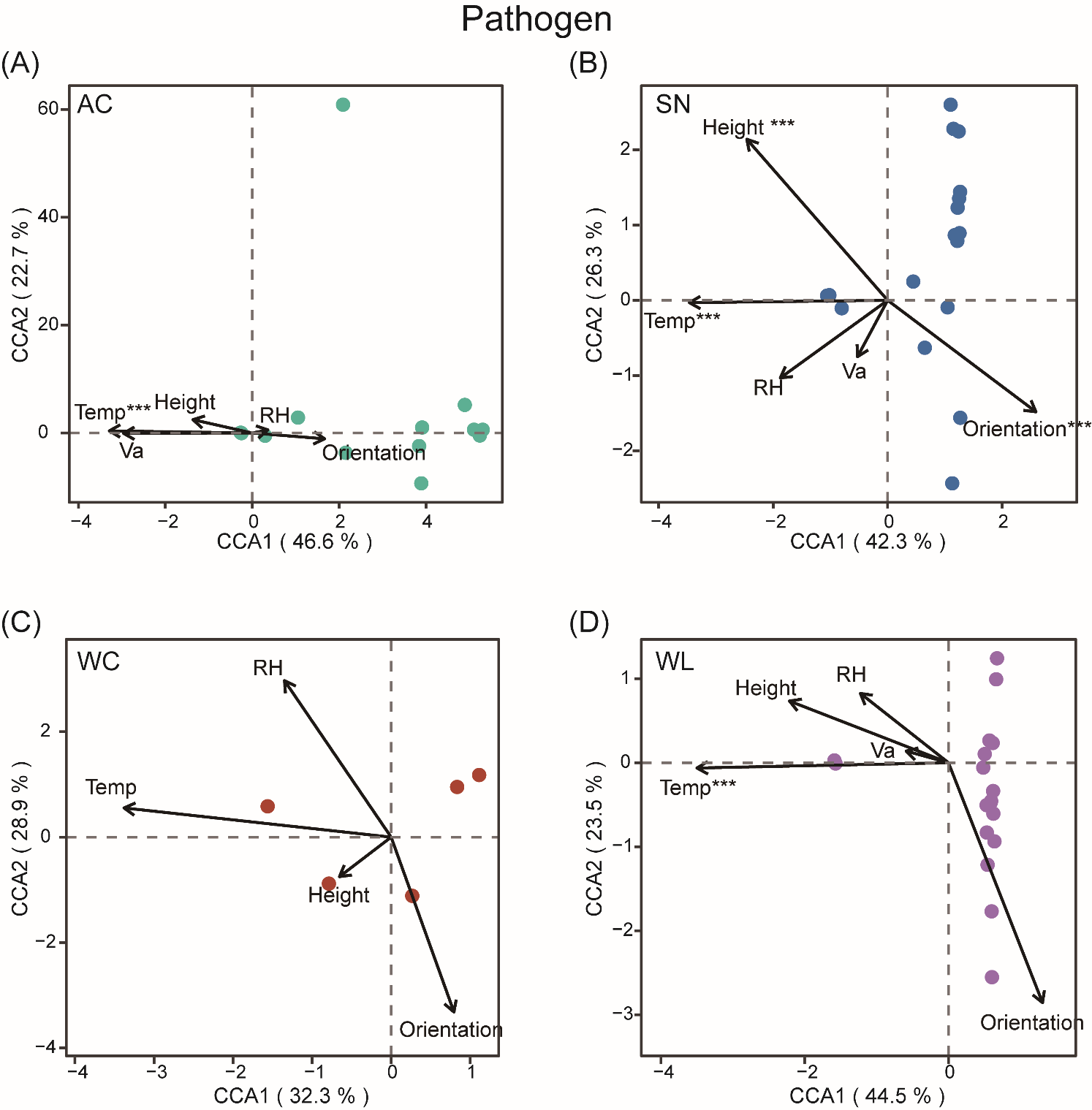


**Supplementary Figure S14.** Canonical correspondence analysis (CCA) of pathogen communities across functional areas. **(A)** AC, **(B)** SN, **(C)** WC, **(D)** WL.


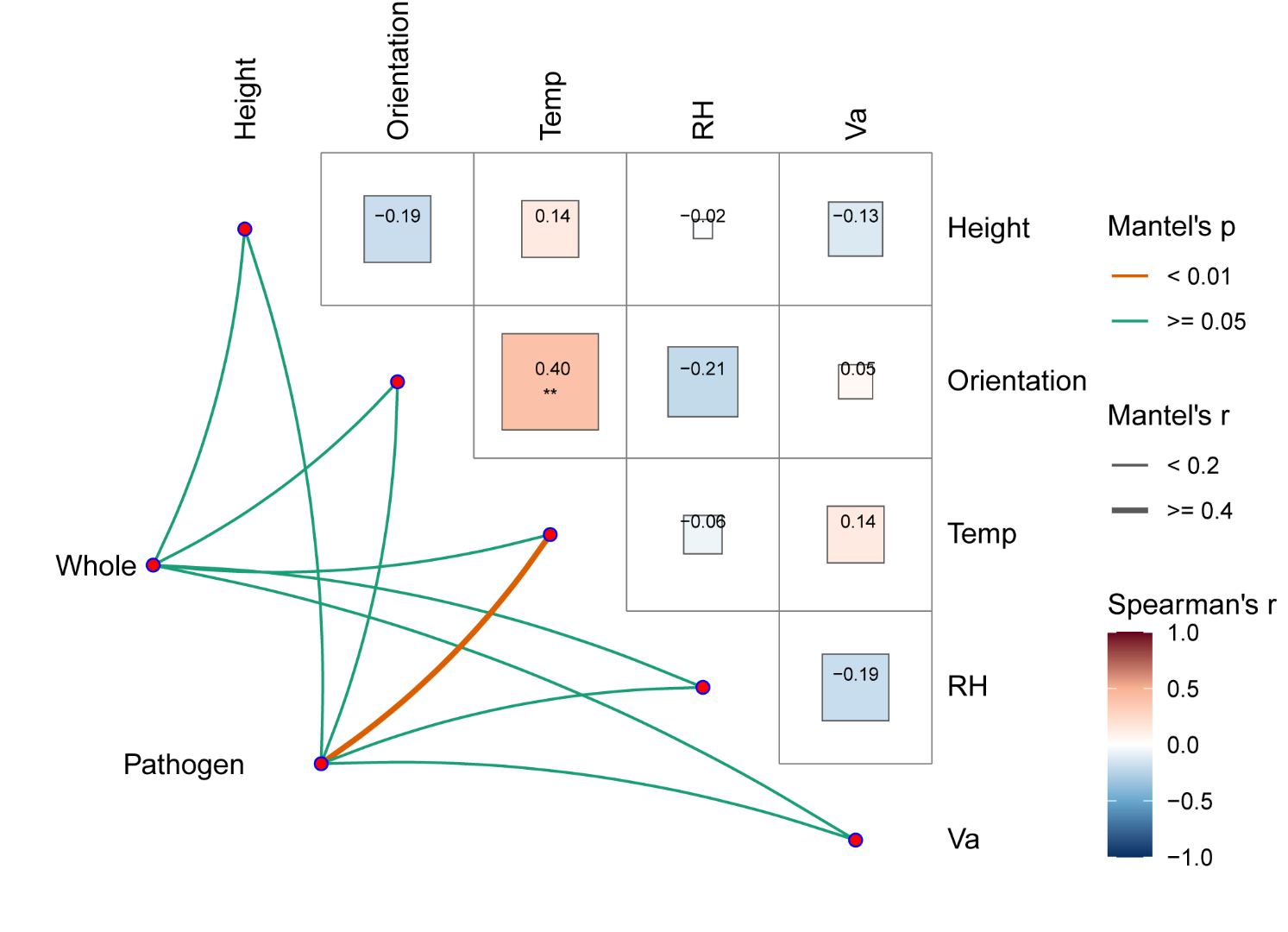


**Supplementary Figure S15.** Mantel test results showing correlations between environmental factors and both the overall bacterial community and potential pathogen communities in the dormitory.


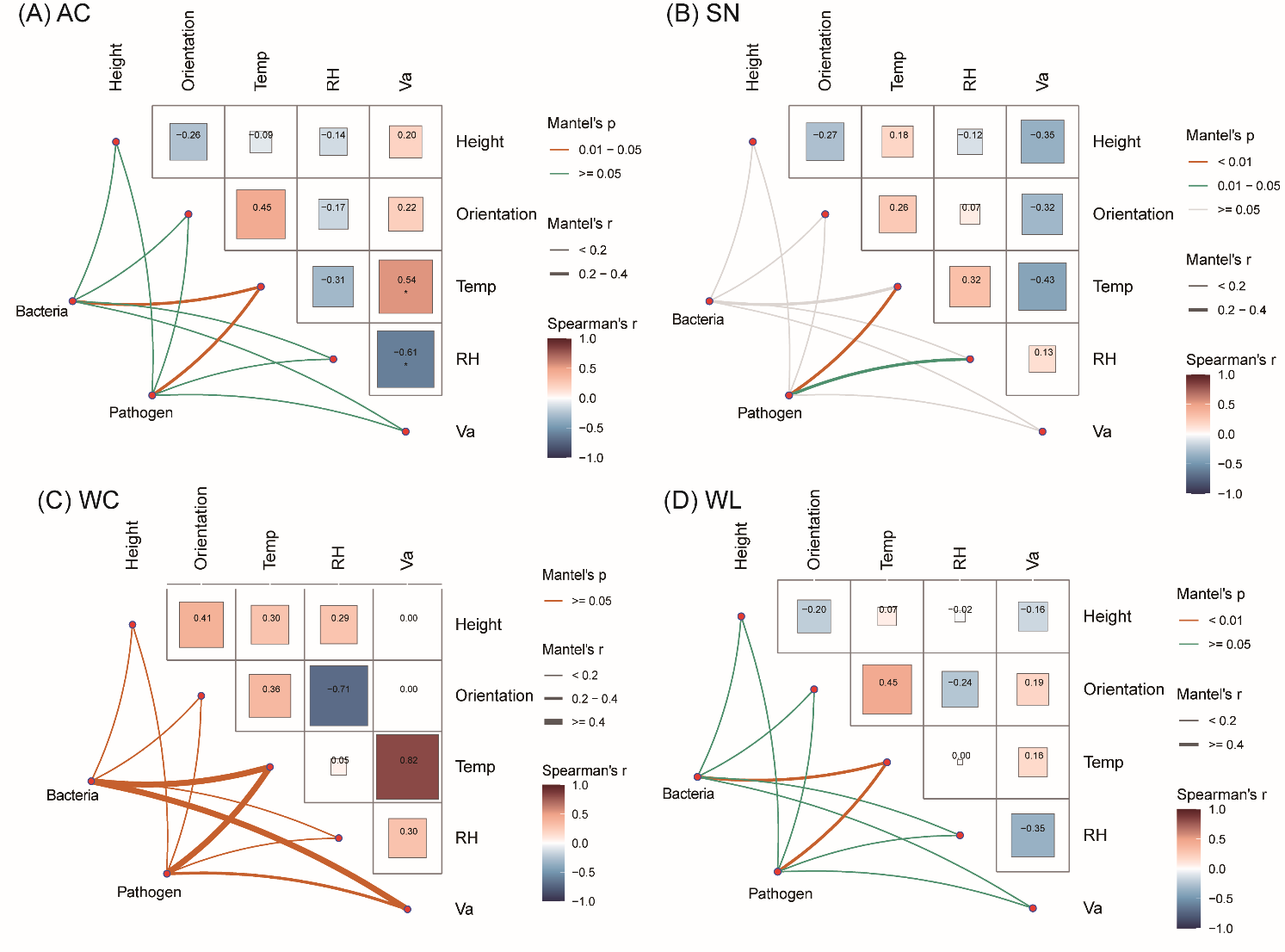


**Supplementary Figure S16.** Mantel test results showing correlations between environmental factors and both the overall bacterial community and potential pathogen communities across functional areas. **(A)** AC, **(B)** SN, **(C)** WC, **(D)** WL.

**

**

**Supplementary Figure S17.** Co-occurrence network analysis of bacterial communities, showing networks for **(A)** the overall bacterial community **(B)** and potential pathogen communities, with nodes colored by module.


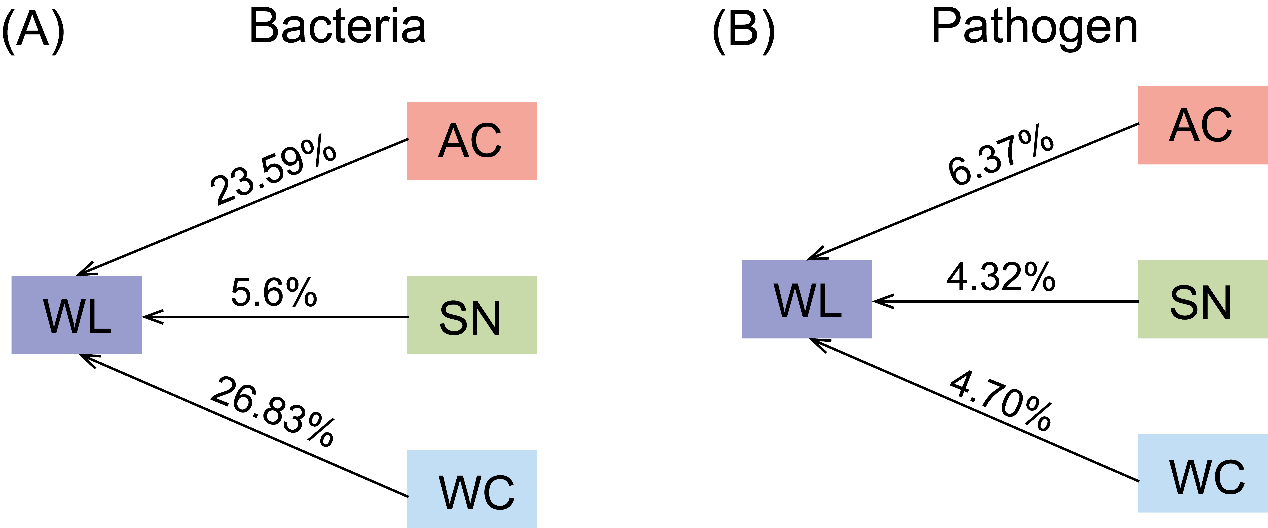


**Supplementary Figure S18.** Source tracking analysis of bacterial communities, including **(A)** the overall bacterial community and **(B)** potential pathogen communities.
